# System with Thermal Management for Synergistic Water Production, Electricity Generation and Crop Irrigation

**DOI:** 10.1007/s40820-025-01876-0

**Published:** 2025-09-03

**Authors:** Meng Wang, Zixiang He, Haixing Chang, Yen Wei, Shiyu Zhang, Ke Wang, Peng Xie, Rupeng Wang, Nanqi Ren, Shih-Hsin Ho

**Affiliations:** 1 State Key Laboratory of Urban-rural Water Resource and Environment, School of Environment, Harbin Institute of Technology, Harbin, 150040 People’s Republic of China; 2 School of Resources & Environmental Science, Hubei Key Laboratory of Biomass-Resources Chemistry and Environmental Biotechnology, Wuhan University, Wuhan, 430079 People’s Republic of China; 3 Department of Chemistry, Tsinghua University, Beijing, 100084 People’s Republic of China

**Keywords:** Thermal management, Water/electricity cogeneration, Cultivation, Water–energy–food nexus, Sustainable development

## Abstract

Sustainable water, energy and food (WEF) supplies are the bedrock upon which human society depends. Solar-driven interfacial evaporation, combined with electricity generation and cultivation, is a promising approach to mitigate the freshwater, energy and food crises. However, the performance of solar-driven systems decreases significantly during operation due to uncontrollable weather. This study proposes an integrated water/electricity cogeneration–cultivation system with superior thermal management. The energy storage evaporator, consisting of energy storage microcapsules/hydrogel composites, is optimally designed for sustainable desalination, achieving an evaporation rate of around 1.91 kg m^−2^ h^−1^. In the dark, heat released from the phase-change layer supported an evaporation rate of around 0.54 kg m^−2^ h^−1^. Reverse electrodialysis harnessed the salinity-gradient energy enhanced during desalination, enabling the long-running WEC system to achieve a power output of ~0.3 W m^−2^, which was almost three times higher than that of conventional seawater/surface water mixing. Additionally, an integrated crop irrigation platform utilized system drainage for real-time, on-demand wheat cultivation without secondary contaminants, facilitating seamless WEF integration. This work presents a novel approach to all-day solar water production, electricity generation and crop irrigation, offering a solution and blueprint for the sustainable development of WEF.

**Supplementary Information:**

The online version contains supplementary material available at 10.1007/s40820-025-01876-0.

## Highlights

- Dynamic thermal management: the system achieves evaporation rates of 1.91 kg m^−2^ h^−1^ (1 sun) and 0.54 kg m^−2^ h^−1^ (darkness) through energy storage hydrogel-based energy storage evaporator, effectively mitigating intermittent solar availability.
- Enhanced salinity gradient utilization: integrated reverse electrodialysis (RED) system harvests ~0.30 W m^−2^ from desalination-concentrated brine, tripling the output of conventional seawater/surface water RED system.
- Sustainable resource integration: drainage water enables zero-pollution crop irrigation (shoot length ~87 mm, 7 d), completing the seamless integration of water-energy-food nexus.

**Supplementary Information:**

The online version contains supplementary material available at 10.1007/s40820-025-01876-0.

## Graphical abstract

**Figure:**
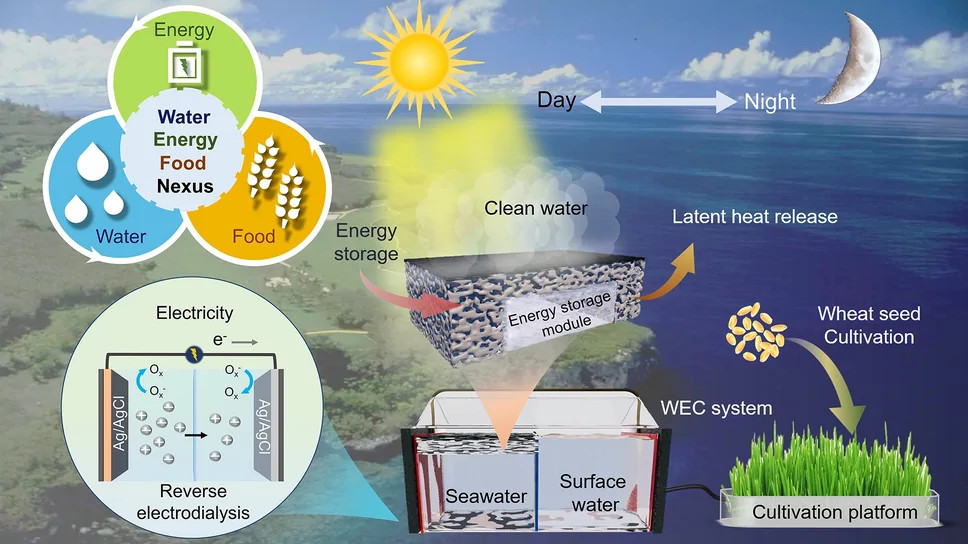

## Introduction

Water, energy and food (WEF) are intertwined and essential elements for sustainable development of human societies, encapsulating three (Goals 2, 6 and 7) of the 17 United Nations Sustainable Development Goals (SDGs) [1, 2]. However, current climate change is exacerbating global water and electricity shortages while also posing challenges to the sustainability of food systems [3, 4]. It is estimated that 6 billion people could be affected by clean water and energy shortages by 2050, with a 70% increase in food production required to feed the world’s population [5–7]. Therefore, we should prioritize the development of sustainable and clean energy to meet global demands, which is essential for addressing the multifaceted issues associated with climate change and ensuring a sustainable future [8].

Solar energy as a green source has received tremendous attention for its ability to generate both fresh water and energy [9–12]. Solar-driven interfacial evaporation is an emerging desalination technology that holds promise for solving water shortage [13–17]. Significant advances have been made in the development of solar evaporators through bionics [18, 19], 3D printing [20, 21], microfluidics [22, 23] and physical/chemical cross-linking gel technologies [24–26]. However, solar intensity-dependent evaporators typically operate intermittently due to the day-night cycle [27]. As a result, evaporator performance would drop sharply by up to 60% under lower light intensity [28, 29]. Furthermore, under normal light condition, the heat generated by the evaporator is inevitably dissipated into the surroundings at lower temperature, thus resulting in low thermal energy utilization [30]. Consequently, there is an urgent need to develop evaporators with advanced thermal management capabilities to address practical challenges caused by variations in solar intensity [31–33].

In addition, the process of solar desalination extracts not only clean water but also salinity-gradient energy from the ocean [34, 35]. In this regard, reverse electrodialysis (RED) technology enables the efficient conversion of the Gibbs free energy (ΔG) from this process into electrical energy [36, 37]. Therefore, effective thermal management during operation is necessary to maintain the durability of desalination and optimize output [38, 39]. At the same time, based on the WEF nexus [40, 41], various components of the water cycle network need to be regulated to achieve efficient outputs (clean water and electricity), while system drainage is optimized for crop irrigation to establish a sustainable agricultural food system. This strategy, which integrates solar, ocean and terrestrial energy, addresses the trade-offs between water, energy, agriculture and climate change. It provides a sustainable supply of materials and energy to regions facing clean water, energy shortages and irrigation water scarcity, significantly enhancing the linkages and interactions between energy security and human well-being.

**Fig. 1:**
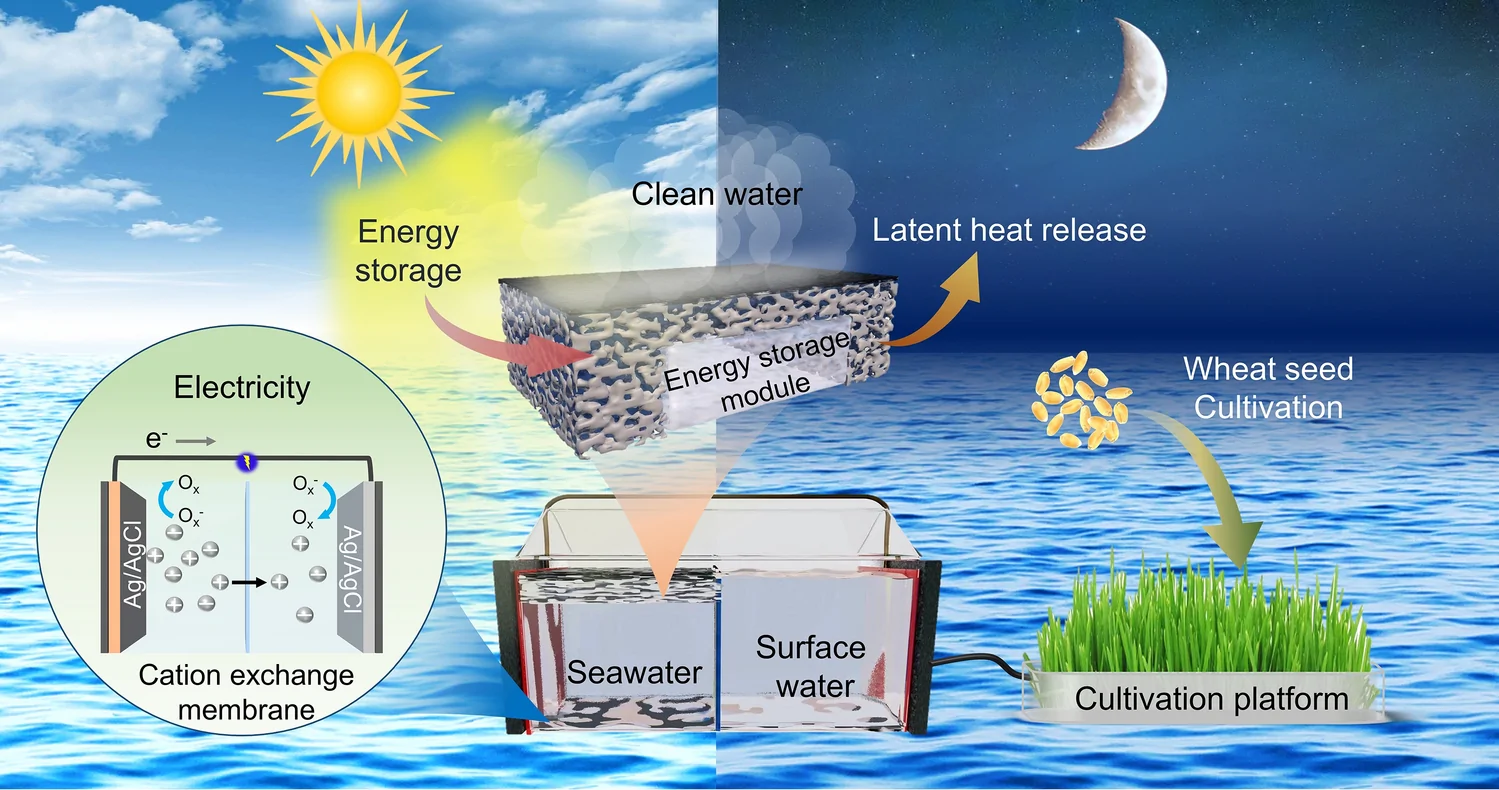
Schematic diagram of the integrated WEC system with energy management based on the WEF nexus. By incorporating an energy storage module inside the evaporator, a high-efficiency evaporator with a thermal management function was created to enable energy storage and latent heat release. During desalination, the increased salt differential energy (*ΔG*_*mix*_) was harvested using RED. Through an integrated crop cultivation platform, drainage from WEC system was utilized for crop irrigation without secondary pollutants

Here, a scalable and efficient all-day integrated system for synergistic water production, electricity generation and crop irrigation (WEC) was developed using a thermal management solar evaporator (Fig. 1). Specifically, polyvinyl alcohol (PVA) hydrogels incorporating energy storage microcapsules (ESMs) were integrated into the evaporator as heat storage modules. This enhances seawater salinity, and RED was employed to efficiently extract salinity-gradient energy in the form of electricity. As a result, WEC system delivers high energy output during continuous operation. Additionally, the system achieves a favorable dark evaporation rate, extending the efficient power generation of RED. The system’s drainage can be used for agricultural irrigation, enabling seamless integration of the WEF nexus without secondary pollution. This technology offers a practical solution to address the global scarcity of water, energy and food, providing a pathway for achieving green and sustainable development.

## Experimental Section

### Materials

Ethanol (AR, 95%), polyvinyl alcohol (PVA, Mw 89,000-98,000) and glutaraldehyde (50%, AR in H_2_O) were purchased from Shanghai Aladdin Biochemical Co., Ltd. (China). HDTMS (hexadecyltrimethoxysilane) was supplied by NanJing ChenGong Organic Silicone Material Co., Ltd. (China). Acetic acid (AR, 99.5%) was purchased from Tian in Fuyu Fine Chemical Co., Ltd. (China). PDMS (Sylgard 184) and the corresponding curing agent were obtained from Dow Corning Co., Ltd. (USA). The cation exchange membrane (CEM) Nafion 117 was purchased from Dupont (USA). The melamine sponge (MS) was purchased from Shanghai Junhua Co., Ltd. (China). All chemicals were used as received without further purification.

### Preparation of ESH, ESE and WEC System

#### Preparation of ESH

For the preparation of ESH, 5 g of PVA (polyvinyl alcohol) is added to 50 mL of deionized water and heated at 90 °C for 1.5 h. Then, 5 g of energy storage microcapsules (ESMs) is added to the solution and stirred thoroughly. Next, 2 mL of 5% glutaraldehyde solution and 5 mL of HCl solution (1 M) are added separately. Finally, the mixture is mixed homogeneously and poured into a mold with dimensions of 3 cm × 3 cm × 1 cm. The mixture is ultrasonicated for 10 min, and the energy storage hydrogel (ESH) is obtained after cooling and curing.

#### Preparation of ESE

Bio-graphene (BG) is prepared based on the previous literature [42]. First, 2 g of bio-graphene (BG) is added to 50 mL of hexane, stirred, and ultrasonically dispersed for 20 min to form a homogeneous suspension. Next, 2 g of HDTMS (Hexadecyltrimethoxysilane) and acetic acid (0.34 g) are added to the BG suspension and stirred for 3 h. PDMS (3.2 g) and curing agent (0.32 g) are then added to the solution and dispersed by ultrasonication for 20 min. The melamine sponge (MS) with original dimensions of 11 cm × 7 cm × 4 cm is cut into blocks of 12, 14, 16 and 18 mm thickness and sonicated in ethanol for 10 min to remove impurities. Using an airbrush (0.3 mm nozzle, 0.2 MPa pressure), 10 mL of the aforementioned suspension was precisely sprayed onto the MS surface from a distance of 10 cm, applying three layers with 30 min drying intervals between layers, followed by thermal curing at 60 °C for 3 h to establish interfacial bonding, resulting in the photothermal layer. Finally, the photothermal layer was cut to the size that could be embedded into the ESH, and the ESH was embedded into the photothermal layer to obtain the energy storage evaporator (ESE).

#### Crop Cultivation Process

For the crop cultivation process, 50 g of wheat seeds is soaked in 200 mL of the corresponding liquid (seawater, surface water, and drainage) for 12 h. The seeds, which had absorbed the liquid sufficiently, are then evenly distributed on the well plates of the planting platform without overlapping to ensure enough space for growth. The surface of the seeds is covered with paper moistened with the corresponding liquid to maintain a moist environment until the average shoot length reaches 10 mm. Irrigation of the wheat is then continued with drainage, seawater and river water, respectively. Throughout the cultivation period, the conditions remained consistent, with room temperature at approximately 22 °C and humidity around 45%. The shoot and root lengths of the wheat are measured daily to accurately track the growth performance.

#### Components of the System

WEC system operates with solar energy input, collects clean water by ESE, extracts energy from the desalination through RED and incorporates a cultivation platform. The system investigated in this study consists of three components: an ESE-based evaporation unit, a RED-based electricity generation unit and a crop cultivation platform utilizing system drainage. The RED component comprises two rectangular half-cells and an ion-selective membrane, with the Nafion membrane (N117) fastened at the junction of the two half-cells. The ESE is positioned within the seawater half-cell, where solar irradiation is applied. Both half-cells are connected to conduits for the respective water supply. The drainage water from the system is collected into the cultivation unit to enable the utilization of drainage water for wheat seed growth.

## Results and Discussion

### Desalination and Thermal Management of the Energy Storage Evaporator (ESE)

**Fig. 2:**
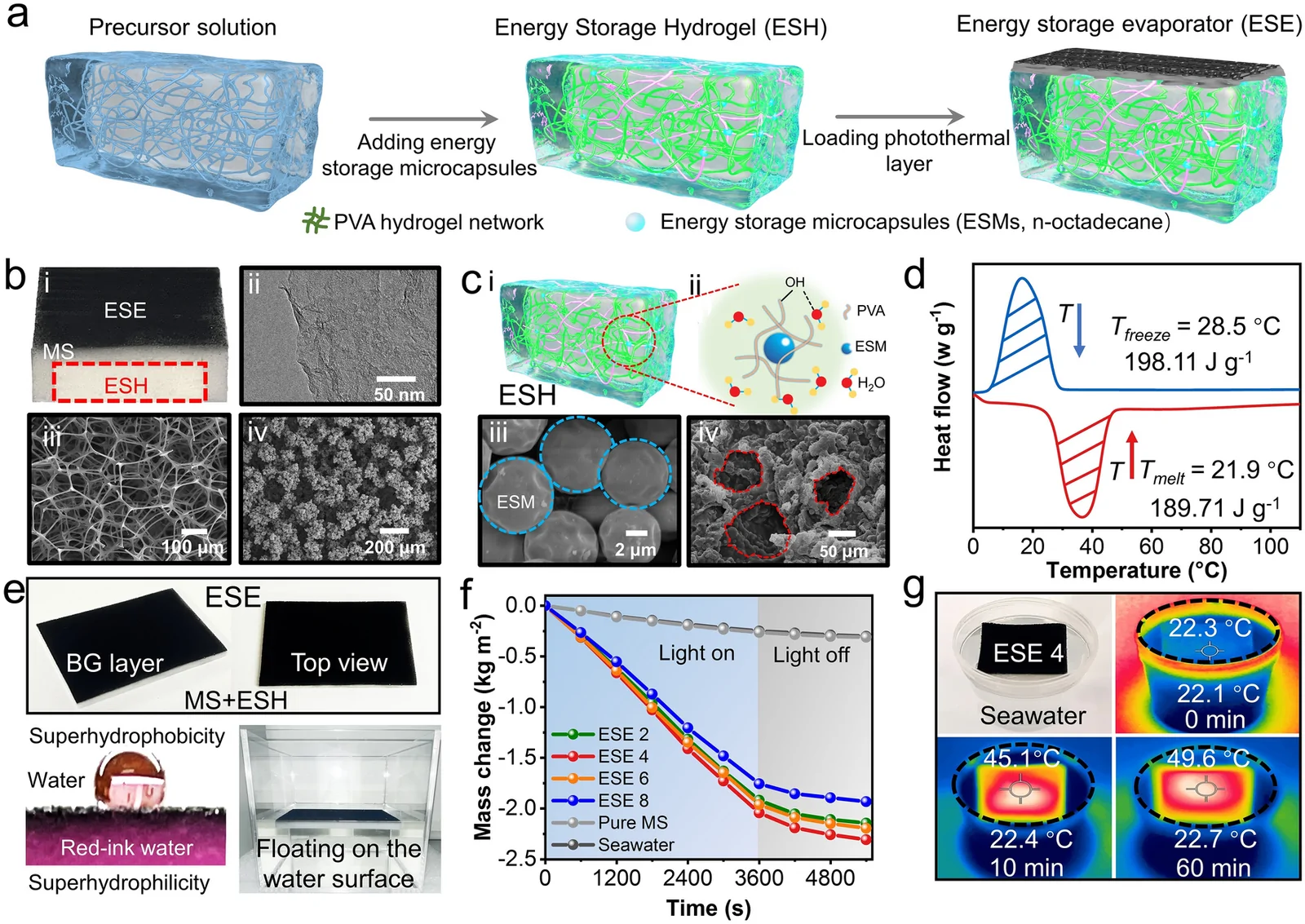
Desalination performance of energy storage evaporator (ESE). **a** Schematic diagram of the preparation process of ESE. **b** Schematic cross section of ESE (i), TEM image of bio-graphene (BG) (ii), SEM images of melamine sponge (MS, iii), and the surface of ESE (iv). **c** Schematic diagram and internal chemical composition of energy storage hydrogel (ESH, i–ii), SEM images of energy storage microcapsules (ESMs, iii), and the internal structure of ESH (iv). **d** DSC curves of ESMs. **e** Optical images of ESE and its state in water. **f** Comparison of mass change in different evaporators (pure MS and ESE 2, 4, 6 and 8) and bulk seawater in sunlight on/off mode. **g** Temperature distribution of ESE 4 and bulk seawater during desalination (60 min, 1 sun)

The energy storage microcapsules (ESMs, n-octadecane) were added to the PVA ([-CH_2_-CHOH-]_n_-) precursor solution and gelatinized to form an energy storage hydrogel (ESH, Fig. 2a). The energy storage evaporator (ESE) was fabricated by embedding the ESH into a photothermal layer made from melamine sponge (MS) and bio-graphene (BG). Industrially produced MS (Fig. S1) is a cost-effective and readily available porous material, and its highly ideal non-aggregate skeletal structure provides excellent access for unrestricted mass transfer [43, 44]. The superhydrophilicity of MS enables rapid water absorption and wetting (Fig. S2), making it an ideal substrate for the evaporator. The cross-sectional structure of the ESE is shown in Fig. 2b-i. Fig. 2b-ii shows the lattice structure of BG, and the surface of BG exhibited superhydrophobicity (WCA=153.2°, Fig. S3). Additionally, the surface of ESE skeleton (Fig. 2b-iii) was encapsulated by BG, exhibiting favorable bonding (Fig. 2b-iv). The chemically cross-linked PVA network forms a hydrophilic polymeric matrix that facilitates rapid water transport and efficient vapor diffusion. The composite structure incorporates ESMs uniformly dispersed within the cross-linked PVA matrix (Fig. 2c-i). The PVA framework physically encapsulates individual ESMs, with surface hydroxyl groups establishing hydrogen-bonding interactions with adjacent water molecules (Fig. 2c-ii). The embedded ESMs provide controlled thermal energy management, exhibiting spherical morphology (average diameter~6 μm) at ambient conditions. Within this hydrogel composite, the porous architecture features interconnected channels (diameter~60–100 μm, Fig. 2c-iv) that enable unimpeded vapor transport during evaporation while simultaneously functioning as a thermal energy reservoir through reversible phase transitions. The phase-change properties of the hydrogel originate from the embedded energy storage microcapsules (ESMs), as evidenced by differential scanning calorimetry (DSC) measurements showing that melting enthalpy (ΔH) = 189.7 J g^−1^, crystallization enthalpy (ΔH) = 198.1 J g^−1^, the phase transition temperature (T_melt_) = 21.9 °C, crystallization temperature (T_freeze_) = 28.5 °C, which demonstrates an optimal match with seawater temperature fluctuations, enabling energy storage and release during operation. Also, the ESE can remain suspended in seawater due to its superhydrophobic surface and hydrophilic interior (Fig. 2e).

The evaporation performance of ESE was evaluated under 1 sun irradiation (AM 1.5G) and dark conditions. The mass changes of bulk seawater, pure MS, ESE 2, ESE 4, ESE 6 and ESE 8 (where 2, 4, 6 and 8 represent the thickness of MS between the surface of ESH and ESE in mm) were compared. As shown in Fig. 2f, the mass change was linear over time, decreasing when under dark conditions. Infrared images (Fig. 2g) show that the surface temperature of ESE 4 rapidly increased to a steady state of 49.6 °C within 60 min, which was significantly higher than the temperature of bulk seawater (22.7 °C). The surface temperatures of ESE 4 and the evaporator without ESH were 49.6 and 49.8 °C, respectively (Fig. S4), indicating that ESH had no effect on the surface temperature of ESE and that thermal energy could be successfully stored in ESMs (T_melt_=21.9 °C).

### Sustainable Water and Electricity Generation Based on Desalination

**Fig. 3:**
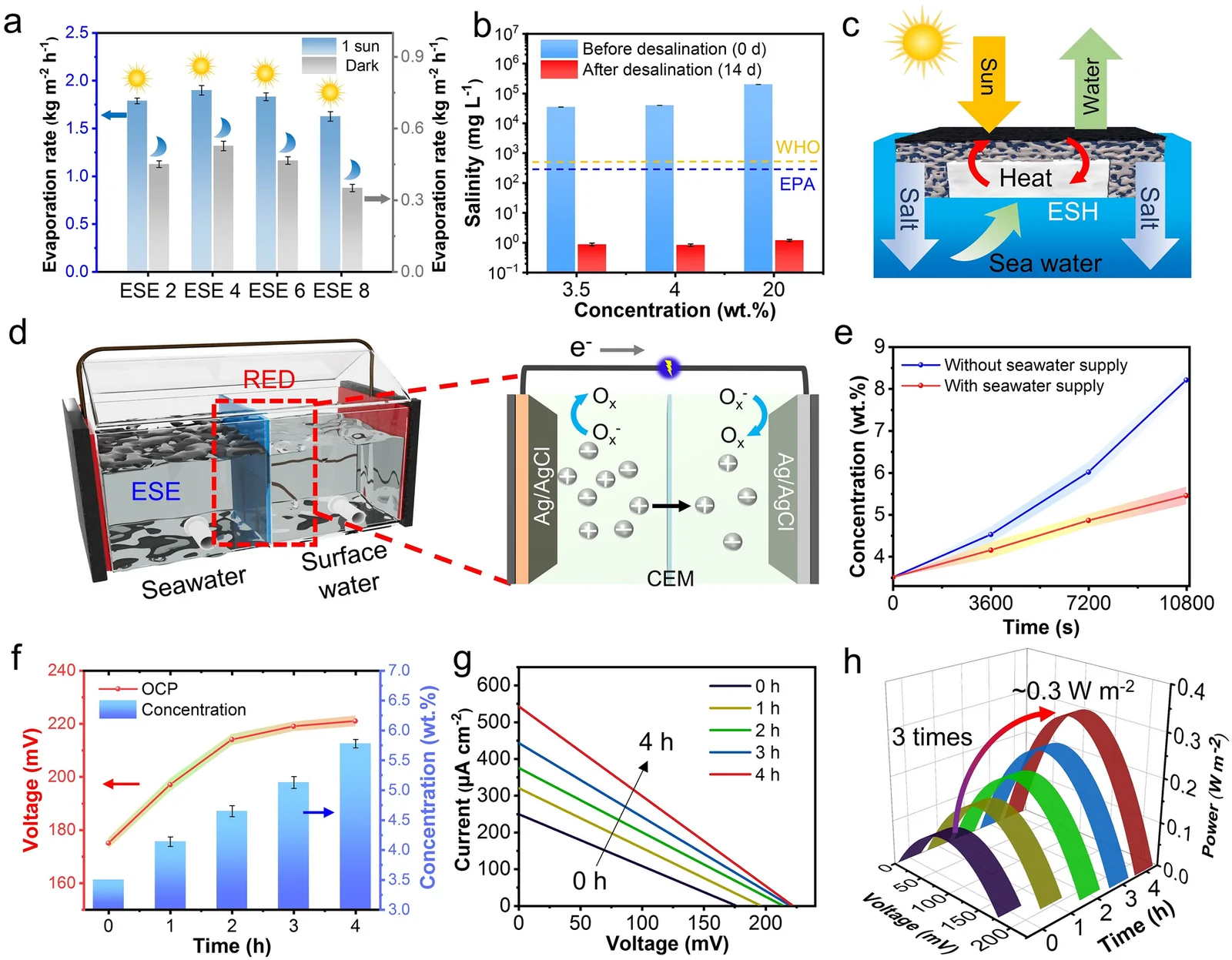
Evaluation of the sustainable electricity generation process based on desalination. **a** Light/dark evaporation rates for different evaporators. **b** Measured salinities of the three samples before and after desalination (14 d). **c** Schematic diagram of evaporation, energy storage processes and salt transport during operation. **d** RED and the mechanism of salt differential energy extraction. **e** Variations in seawater concentrations with and without seawater supply. **f** Variations in open-circuit voltage (*E*_*oc*_) and concentration after RED reached steady state (within 4 h). **g** I–V curve of RED under seawater desalination. **h** *P*_*max*_ of RED from operation to steady state

ESE 4 exhibited the optimum evaporation rate (~1.91 kg m^−2^ h^−1^), whereas ESE 2, 6 and 8 had evaporation rates of ~1.78, ~1.83 and ~1.62 kg m^−2^ h^−1^, respectively (Fig. 3a, Note S1). This difference was attributed to the varying absorption of surface thermal energy by the seawater contained in the MS layer with different thicknesses. In contrast, the lower evaporation rates of bulk seawater and pure MS were due to the lack of integration with photothermal conversion in their desalination processes. The evaporation rates at different solar intensities are shown in Fig. S5. Additionally, ESE 4 maintained a dark evaporation rate of ~0.53 kg m^−2^ h^−1^, attributed to the heat released by ESH under dark conditions (Note S2). The ESE demonstrated exceptional thermal cycling stability over 30 cycles, with peak surface temperature maintained at ~49.7 °C and sustained temperature at ~27.6 °C, without observable performance degradation (Fig. S6a). The mass of the ESE remained unchanged during the thermal cycling process (~15.28 g, Fig. S6b), confirming the excellent thermal cyclic performance of ESE. Moreover, the ESE was tested for 21 d in a continuous long-term cycling (Fig. S7) and the evaporation rate was maintained at ~1.90 kg m^−2^ h^−1^; the SEM (inset) showed undamaged internal structure after 21 d of cycling, demonstrating that the ESE has the potential for long-term operation. After compression–recovery cycles (Fig. S8), the stress–strain curve of the ESE maintained exceptional consistency with the initial cycle, with measured compressive strength (*Rmc*) and compressive elastic modulus (*Ec*) reaching ~0.19 and ~1.10 MPa, respectively. These results confirm the excellent elastic recovery capability and robust mechanical properties of ESE. Due to the excellent evaporation performance of ESE (Table S1), the salt concentration of the condensed water collected during desalination at various salt concentrations (ocean (3.5 wt%), Red Sea (4.0 wt%) and near-saturated solution (20 wt%)) remained well below the salinity levels (four orders of magnitude) set by the World Health Organization (WHO) and the US Environmental Protection Agency (EPA) (Fig. 3b). The high evaporation rate of ESE 4 was attributed to the specially constructed evaporator structure (Fig. 3c), showing a photothermal layer can couple photothermal conversion with steam production, and a bottom ESH can store waste heat from the desalination process. The hydrophilic structure of ESE ensures unobstructed channels to achieve continuous water pumping and salt transportation. Finite element simulations further confirm the enhanced salt transport process based on surface thermal localization and salinity gradients, ensuring the continuous desalination capability of the ESE (Note S3, Fig. S9).

For electricity generation, a desalination-based RED system was utilized to evaluate the impact of the desalination process on electricity generation from salt differential energy (Note S4, Fig. S10). Figure 3d presents a typical schematic where a cation exchange membrane (CEM, Nafion 117) was assembled between seawater and surface water. Cations penetrated through CEM and were transported toward the surface water side, driven by the salinity gradient. The desalination process affected the salinity, as shown in Fig. 3e, where the salt concentrations rose from 3.50 wt% (0 s) to ~5.45 and ~8.21 wt% (10,800 s) under conditions with and without seawater supply, respectively. However, in the absence of seawater supply, continuous evaporation ultimately resulted in the saturation and precipitation of seawater salts (Fig. S11), disrupting the system’s functionality. Therefore, maintaining a steady supply of seawater to RED is crucial for preventing declines in seawater levels and salinity saturation, thereby enabling sustainable enhancement of seawater salinity and stabilizing electricity generation efficiency.

As shown in Fig. 3f, the salinity of seawater increased linearly from the initial 3.5 wt% to ~5.78 wt% (4 h). This is accompanied by an increase in the system’s open-circuit voltage (*E*_*oc*_) from an initial value of ~175 mV to a saturation value of ~221 mV. Conversely, the *E*_*oc*_ of RED without the solar desalination process decreased to ~85 mV after 4 h of continuous operation (Fig. S12). These results indicate that the desalination process can increase the *Eoc* of RED system, and the continuous increase in salinity effectively replenishes the reactive species near the Ag/AgCl electrodes. This prevents the efficiency degradation that occurs during RED operation in the absence of solar desalination. Consequently, the integrated desalination-RED system can be synergized for sustainable water production and electricity generation.

In addition, typical current–voltage (I–V) curves and output power density were measured in this investigation (Note S5). The short-circuit current (*I*_*sc*_) of RED was only ~249 μA (*E*_*oc*_ ~175 mV) in the initial condition. As the system continued to operate, the *Eoc* reached a steady value of ~222 mV, and the corresponding *Isc* reached ~543 μA (Fig. 3g). Meanwhile, the power density (*P*_*max*_) in the initial state was ~0.11 W m^−2^. As the operation time increased (4 h, ~222 mV), the *P*_*max*_ stabilized at ~0.3 W m^−2^, nearly three times higher than the initial state (Fig. 3h). In contrast, in the absence of solar desalination process, the *P*_*max*_ continuously declined during operation, reaching a value of ~0.02 W m^−2^ (Fig. S13). These results indicate that the heightened salinity gradient during seawater desalination provides a strong intrinsic driving force for increasing the power density of RED, offering new insights into the sustainable delivery of clean water and electricity.

### All-Day Performance Testing of Water/Electricity Cogeneration

**Fig. 4:**
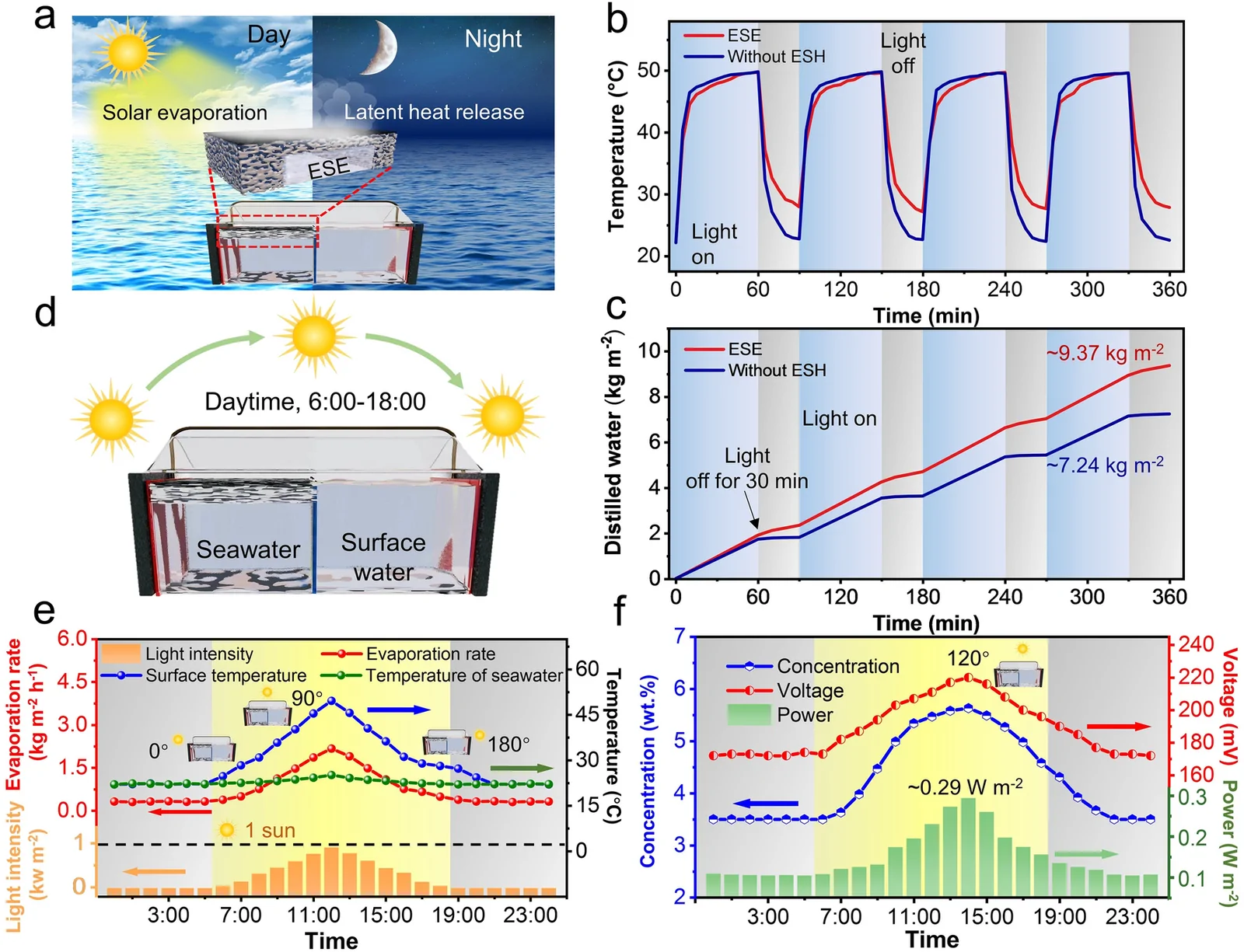
Performance testing of WEC system for all-day water/electricity cogeneration. **a** Schematic of the all-day water/electricity cogeneration system. **b** Variation in the surface temperature of ESE and the evaporator without ESH during four sunlight on/off cycles (AM 1.5G). **c** Mass change of the distilled water in ESE and the evaporator without ESH during four sunlight on/off cycles. **d** Changes in light angle throughout the day. **e** Dynamic variations in light intensity, surface and seawater temperature, as well as the evaporation rate in WEC system under simulated all-day conditions. **f** Dynamic variations in the concentration, voltage (*E*_*oc*_) and power (*P*_*max*_) of WEC system under simulated all-day conditions

To assess the sustainability of clean water/electricity cogeneration, the system (Fig. 4a) integrating ESE and RED was evaluated for all-day operational effectiveness. The surface temperatures during desalination for four light on/off cycles are shown in Fig. 4b, with the evaporator without ESH serving as a control. As expected, the surface temperature distributions of ESE and the evaporator without ESH exhibited no discernible difference under AM 1.5G, with both reaching a maximum temperature of ~49.6 °C. After the light was turned off, the cooling rates of the ESE and the evaporator without ESH were measured at ~0.72 and ~0.91 °C min^−1^, respectively, demonstrating a 27% slower cooling rate for the ESE. Furthermore, upon completion of a single cycle, the ESE surface temperature (~27.9 °C) remained 23% higher than that of the evaporator without ESH (~22.7 °C), which is attributed to the effective release of stored thermal energy from the ESH (Figs. S14 and S15). Furthermore, the linear fit of the mass change curves concerning light duration was well matched. In this case, the water production of ESE (~9.37 kg m^−2^) exceeded that of the evaporator without ESH (~7.24 kg m^−2^) in all four cycles, showing a ~30% increase in total water yield (Fig. 4c). It is noted that the desalination performance of ESE was more efficient in successive light-dark cycles due to continuous evaporation (Fig. S16).

Furthermore, intermittent sunlight irradiation (day and night, Fig. 4d) can affect the desalination process and indirectly influence power generation efficiency, making it necessary to assess desalination and output performance during actual operation. As shown in Fig. 4e, the initial surface temperature of ESE was ~22 °C, which increased to ~49.6 °C (12:00, 1 sun) as the light intensity rose. The evaporation rate was positively correlated with light intensity, peaking at ~1.91 kg m^−2^ h^−1^ (1 sun, 12:00). After, as the light angle gradually increased from 90° to 180°, the surface temperature decreased to ~22.1 °C, and the evaporation rate dropped to ~0.23–0.25 kg m⁻^2^ h⁻^1^. Additionally, the seawater temperature remained relatively stable (22-24.1 °C), demonstrating that the excellent photothermal conversion of ESE.

As shown in Fig. 4f, the salinity of the system increased continuously from ~3.5 wt% to ~5.63 wt% during the period between 6:00 and 14:00 due to varying light intensity. This was resulted in the continuous increase of *Eoc* and *P*_*max*_ from the initial ~173 mV and ~0.11 W m^−2^ to ~220 mV and ~0.29 W m^−2^, respectively. This increase was attributed to the rise in seawater salinity, leading to a gradual increase in electricity generation. Notably, the peak power of the system (14:00) occurred after the peak desalination rate (12:00), as the reduction in evaporation rate between 12:00 and 14:00 slowed the increase in salinity, which had not yet reached its peak (salinity peaked at 14:00, ~5.63 wt%). Moreover, as the light angle increased, system performance from 14:00 to 22:00 exhibited a declining trend, with *Eoc* and *P*_*max*_ decreasing from ~220 to ~172 mV and ~0.29 to ~0.109 W m^−2^, respectively. Although the system switched to night mode after 18:00, the electrodes continued to consume the increased salinity from daytime, delaying the minimum values of *Eoc* and *P*_*max*_ until 21:00. During nighttime, *P*_*max*_ remained at ~0.11 W m^−2^ (21:00-6:00). Given the inherent concentration difference between seawater and surface water, WEC system could operate continuously in darkness [45, 46]. In summary, the system was able to extract high differential salt energy from the desalination process during daytime and natural differential salt energy between seawater and surface water during nighttime. Meanwhile, the power output of the system remained stable during a 30-day cyclic operation, with *P*_*max*_ remaining at ~0.29 W m^−2^ (Fig. S17), indicating favorable stability and long-term operational potential.

### Feasibility of WEC System Drainage for Wheat Cultivation

**Fig. 5:**
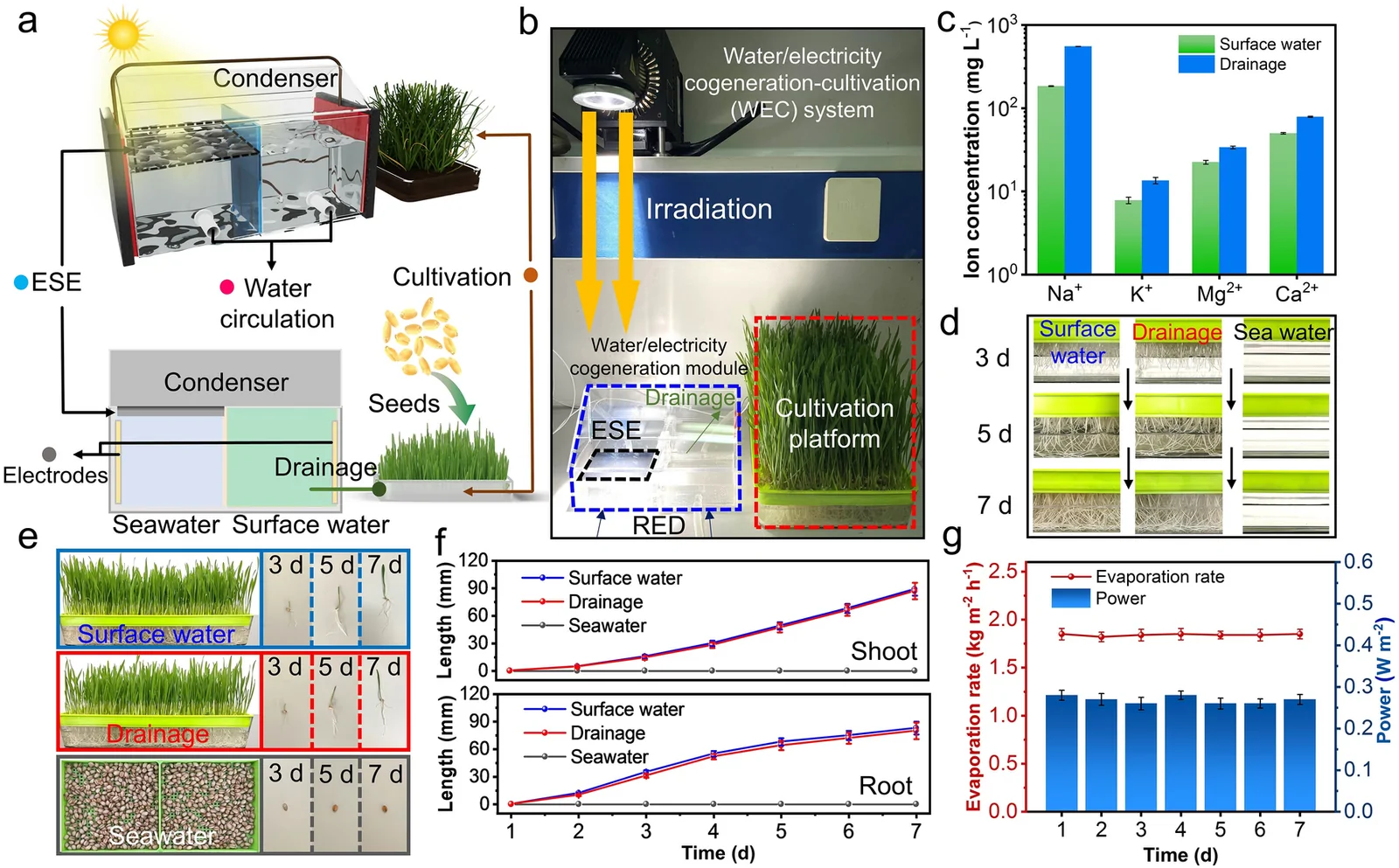
Growth assessment of wheat cultivation using WEC drainage. **a** Schematic illustration of WEC system. **b** Optical image of WEC system; **c**) Drainage ion concentrations (Na^+^, K^+^, Mg^2+^ and Ca^2+^) for the initial and stabilized operation of WEC system. **d** Optical images of wheat root growth (3, 5 and 7 d) irrigated with surface water, WEC drainage and seawater, respectively. **e** Optical images of the growth of wheat seedlings after 3, 5 and 7 d of irrigation with surface water, WEC drainage and seawater, respectively. **f** Shoot and root lengths of wheat seedlings irrigated with surface water, WEC drainage and seawater, related to the cultivation period. **g** Evaporation rate and power output of WEC system over the wheat cultivation period

Based on a sustainable WEF nexus, a comprehensive water cycle should include clean water, electricity production and crop irrigation to maximize the use of water resources [47]. As evidenced by the preceding analysis, the ESE-based RED system demonstrates optimal performance under all weather conditions. Therefore, a rational water recycling network should be constructed based on the WEF nexus, utilizing system drainage for crop irrigation (Fig. 5a). Detailed in Fig. 5b, a rational water circulation pathway connects the water/electricity cogeneration module to the agricultural cultivation platform, with drainage being gathered and directed to the platform as needed (Fig. S18). As shown in Fig. 5c, the initial concentrations of Na^+^, K^+^, Mg^2+^ and Ca^2+^ in surface water were 183.7, 7.8, 22.4 and 49.8 mg L^−1^, respectively. After the system stabilized, the evaporation rate reached ~1.91 kg m^−2^ h^−1^, and the power output achieved ~0.29 W m^−2^ (Fig. S19). The ionic concentrations of the drainage were Na^+^~552.8 mg L^−1^, K^+^~13.5 mg L^−1^, Mg^2+^~33.7 mg L^−1^ and Ca^2+^~78.8 mg L^−1^, which were significantly lower than those found in Chinese offshore seawater (Fig. S20).

In this work, the crop irrigation capacity of the drainage was investigated using surface water and seawater as controls. Figure 5d depicts the evolution of root length in seeds irrigated with surface water, drainage and seawater over periods of 3, 5 and 7 d, respectively. It is noted here that seawater-irrigated wheat seeds did not root throughout the growth cycle, as the salt concentration exceeded the germination tolerance threshold for wheat (Fig. S21) [48, 49]. The root system of wheat was irrigated with surface water and drainage water. As the incubation process proceeded, the white roots transitioned from a sparse state at 3 d to tightly intertwined at 7 d, with the robust and well-developed root system promoting the full absorption of nutrients by the wheat seedlings [50].

Similar to root growth, wheat seeds irrigated with seawater did not germinate (Fig. S22). On the other hand, wheat irrigated with drainage exhibited a similar trend in shoot length over time to that of wheat irrigated with surface water (Fig. 5d). This demonstrates the excellent compatibility between the system drainage and crop irrigation, meeting the actual needs of wheat throughout the cultivation cycle. In this investigation, shoot and root lengths of wheat cultivation were recorded separately (Fig. 5e). During the cultivation cycle, it is noted that the shoot and root lengths of wheat irrigated with drainage and surface water had comparable growth trends, reaching ~87 and ~89 mm (shoot length), as well as ~80 and ~83 mm (root length) within 7 d, respectively. Meanwhile, the steady-state WEC system had a drainage collection rate of 12 L m^−2^ h^−1^ (Fig. S23), which satisfied the water demand for crop cultivation. Additionally, throughout the entire cultivation period, the evaporation rate and *P*_*max*_ of WEC system were maintained at ~1.89 kg m^−2^ h^−1^ and ~0.29 W m^−2^, respectively (Fig. 5f). This demonstrates that the system is both feasible and self-sustaining.

### Carbon Offsets Provided by the Operation of WEC System

**Fig. 6:**
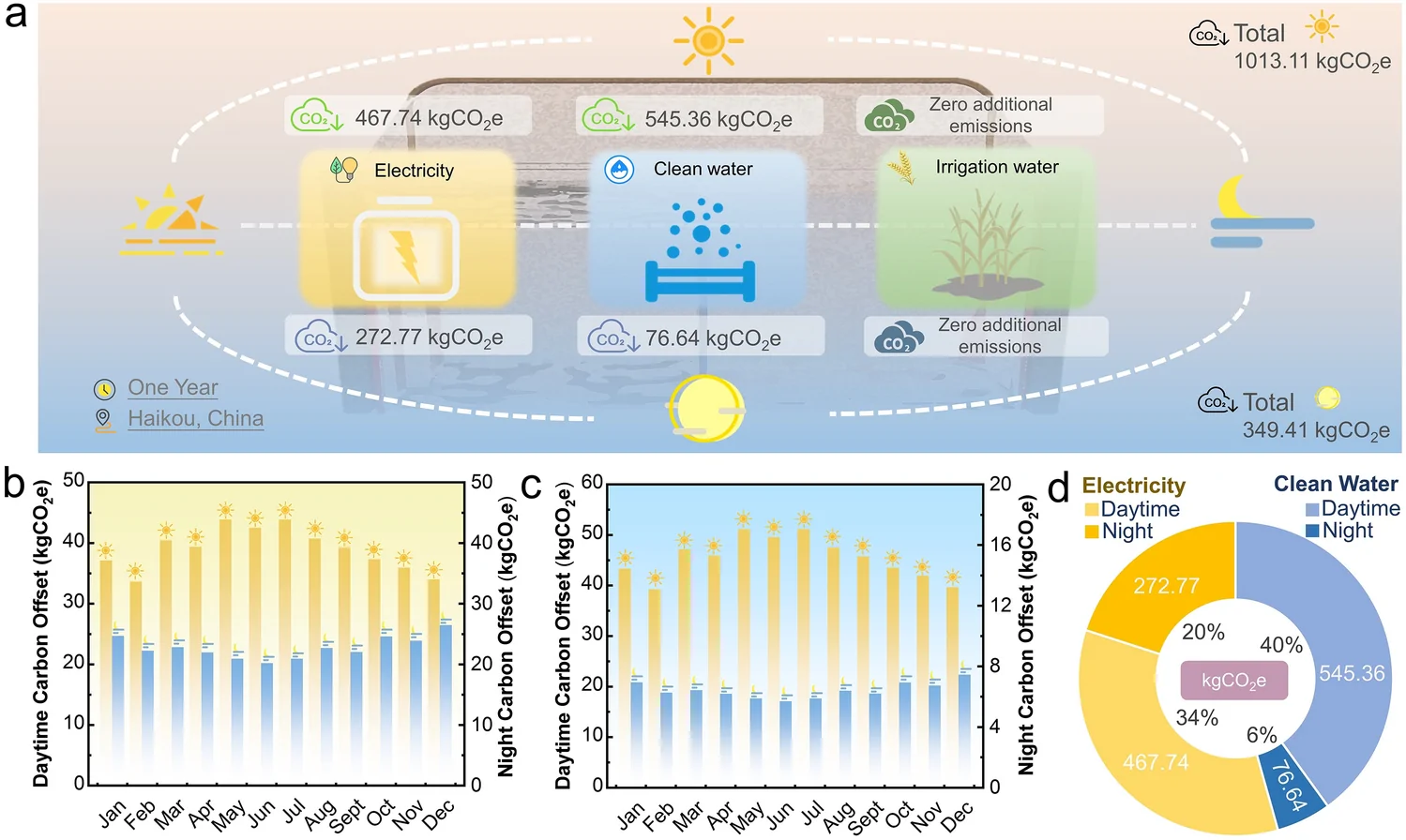
Carbon offsets generated by the operation of WEC system. **a** Carbon offsets achieved through the generation of electricity, clean water and irrigation water by WEC system over the entire assessment period. **b** Monthly carbon offsets resulting from electricity production by WEC system, covering both daytime and nighttime operations. **c** Monthly carbon offsets achieved through clean water production by WEC system, covering both daytime and nighttime operations. **d** Carbon offset ratio derived from electricity and clean water production by WEC system over the entire assessment period

To provide a pathway for achieving carbon neutrality, a preliminary assessment of the carbon offsets provided during the operational cycle of WEC system has been conducted (Note S6, Figs. S24–S29). As illustrated in Fig. 6a, the assessment specifically evaluated the greenhouse gas (GHG) offsets generated by the system’s integrated processes of electricity generation, clean water production and irrigation water supply. Over a one-year operational period, the system has demonstrated the capability to offset a total of 1,362.52 kgCO_2_e (kilograms of CO_2_ equivalent) in GHG emissions, which is approximately equivalent to offsetting the GHG emissions produced by combustion of 1,172 m^3^ of natural gas. Additionally, it is noteworthy that the process of supplying irrigation water does not contribute to additional carbon emissions in agricultural production.

As depicted in Fig. 6b, throughout the operational cycle of WEC system, the carbon offsets associated with electricity generation are notably higher during daytime compared to nighttime. The period from May to August exhibits the pinnacle of these carbon offsets. Nighttime carbon offsets primarily stem from RED power generation facilitated by thermal management. Specifically, the integration of thermal management in the evaporator enhances carbon offsets by an additional 26%, accumulating to a total of 349.41 kgCO_2_e. The monthly trends in carbon offsets from clean water production generally align with those of electricity generation, as illustrated in Fig. 6c, with May through August marking the peak offset months within the operational cycle. This alignment is attributable to the heightened solar intensity and extended daylight hours during summer, which bolster the system’s capacity to generate electricity, clean water and irrigation water more efficiently than during winter, consequently leading to the augmented carbon offsets (Fig. 6d). In terms of proportion (Fig. 6e), electricity generation accounts for 54% of the carbon offsets, whereas clean water production constitutes 46%. Due to the distinct attributes of these products, the carbon offsets derived from electricity generation marginally surpass those from clean water production by approximately 8%, equating to 740.51 and 622.0 kgCO_2_e, respectively. Upon preliminary assessment, it is evident that WEC system exhibits sustained carbon offsetting capabilities throughout the entire day, thus holding substantial promise for attaining carbon neutrality.

The operation mode of water production, electricity generation and crop irrigation possesses high energy utilization efficiency (Note S7, Fig. S30). Through WEC system, sunlight can be highly converted into thermal energy through effective absorption and release of latent heat by ESE with thermal management capabilities. This activates water molecules to produce an efficient interfacial evaporation effect. The enhanced water evaporation can increase the salinity difference between seawater and surface water, with the energy generated from this concentration difference often overlooked in previous studies. The advantage of capturing energy through RED system is that the salinity gradient can be directly utilized to generate electricity. Moreover, WEC drainage can be effectively optimized for agricultural applications (Fig. S31). Outdoor experiments demonstrated the excellent operational feasibility of the WEC (Note S8, Fig. S32), which is exemplifying a sustainable model that seamlessly integrates solar desalination, electricity generation and crop cultivation within the WEF nexus framework. Approaches to enhance system efficiency include but are not limited to: 1) constructing hybrid photothermal evaporators with hierarchical water channels; 2) employing broadband metamaterials to increase light absorption beyond 95%; 3) integrating aerogel insulation layers at the evaporator base to suppress conductive heat loss; 4) utilizing thinner ion-exchange membranes to reduce membrane resistance. Optimizing key components such as water transport pathways, photothermal conversion efficiency, thermal management modules and ion-selective membranes holds significant potential for substantially improving overall system performance [51–54]. This approach enhances resource efficiency and sustainability, demonstrating a holistic solution that addresses critical challenges in water, energy and food security.

## Conclusions

In this work, a sustainable water–energy–food (WEF) nexus is demonstrated through the design of an integrated WEC system with advanced energy management capabilities. The meticulously designed ESE exhibits high solar absorptivity, excellent photothermal conversion and superior heat storage and release properties. The ESE-based WEC system effectively extracts clean water and harnesses high differential salt energy produced during desalination, while system drainage is optimized for crop cultivation. By utilizing both ESE and RED, the system enables desalination and power harvesting, with the evaporation time of ESE system extended by ~1 h (~0.54 kg m^−2^ h^−1^) in the dark, following highly efficient solar desalination (AM 1.5G, ~1.91 kg m^−2^ h^−1^). In addition, WEC system generates excellent electricity (~0.3 W m^−2^) by extracting salt differential energy under all weather conditions. Through its crop cultivation platform, system drainage supports wheat cultivation, with shoot and root lengths reaching ~87 and ~80 mm within 7 d, respectively. The remarkable all-day output of WEC system offers significant potential for low-cost utilization. Carbon offset assessment indicates that WEC system has the capability to neutralize a total of 1,362.52 kgCO_2_e of GHG emissions during its operational cycle. Additionally, WEC system facilitates the integrated use of solar, seawater and land-based energy, paving the way for promising prospects in sustainable development within the WEF nexus. This innovative system with energy management capabilities is expected to have broad applications, including desalination, wastewater treatment, electricity generation and crop cultivation [55, 56], contributing to the realization of global carbon neutrality goals.

## Supplementary Information

Below is the link to the electronic supplementary material.

Supplementary file1 (DOCX 25654 KB)
