# Supplementary material for: System with Thermal Management for Synergistic Water Production, Electricity Generation and Crop Irrigation
Source: Nanomicro Lett. 2025 Sep 3;18:57. doi: 10.1007/s40820-025-01876-0 (PMC12405090; doi:10.1007/s40820-025-01876-0)
Supplement: Supplementary file 1 — Supplementary file1 (DOCX 25654 KB) [file 40820_2025_1876_MOESM1_ESM.docx]

Supporting Information for

**System with Thermal Management for Synergistic Water Production, Electricity Generation and Crop Irrigation**

Meng Wang ^1†^, Zixiang He ^1†^, Haixing Chang ^2†^, Yen Wei ^3^, Shiyu Zhang ^1^, Ke Wang ^1^, Peng Xie ^1^, Rupeng Wang ^1^, Nanqi-Ren ^1^, Shih-Hsin Ho ^1, *^

^1^ State Key Laboratory of Urban-rural Water Resource and Environment, School of Environment, Harbin Institute of Technology, Harbin 150040, P. R. China

^2^ School of Resources & Environmental Science, Hubei Key Laboratory of Biomass-Resources Chemistry and Environmental Biotechnology, Wuhan University, Wuhan 430079, P. R. China

^3^ Department of Chemistry, Tsinghua University, Beijing 100084, P. R. China

^†^ Meng Wang, Zixiang He, and Haixing Chang contributed equally to this work.

* Corresponding author. E-mail: [stephen6949@hit.edu.cn](mailto:stephen6949@hit.edu.cn) (Shih-Hsin Ho)

**S1 Experimental details**

**S1.1 Preparation of homemade Ag/AgCl electrode**

Silver mesh (99.99%, 200 mesh) was cut into 1 cm × 2 cm slices and sonicated in ethanol for 15 min to remove impurities. The positive wire of the DC power supply was connected to the aforementioned cut silver mesh, while the negative wire was connected to a commercial platinum electrode. The silver mesh was then electrochemically oxidized in 0.6 mol L^-1^ NaCl solution at a voltage of 1.8 V for 1.5 min.

**S1.2 Simulated all-day irradiation experiment**

In this study, the light angle (AM 1.5G, 0-180°) was adjusted along a semicircular path to simulate the system’s actual sunlight exposure during daytime (6:00-18:00), and the light was turned off for 12 hours (18:00-6:00) to simulate night period.

**S1.3 Materials characterization**

The structural features of the MS and ESE evaporator were observed using a scanning electron microscope with an accelerating voltage of 15 kV (ZEISS Sigma-500, Germany). High-resolution images of BG were obtained using the Talos F200X, USA. The water contact angle (WCA) was quantified using an OCA20 system (Data-physics, Germany). DSC curves for ESMs were obtained using the Mettler DSC3, Switzerland. Infrared images were taken by an infrared camera (FLIR E8, USA). Ion concentrations were quantified using an inductively coupled plasma mass spectrometer (NexION 350X, USA). Evaporation experiments were conducted under a solar simulator PLS-SXE300 (Perfectlight, China).

**Note S1 Evaporation rate calculation**

The evaporation rate ($ṁ$) is expressed as the rate of change in mass over time ($\frac{d_{m}}{d_{t}}$). Therefore, the calculation formulas for the evaporation rate of ESE:

$\begin{aligned} ṁ=\frac{d_{m}}{S\cdot d_{t}}\#\left( S1 \right) \end{aligned}$

where $d_{m}$ denotes the mass change by using EUE, $S$ is the evaporation area, and $d_{t}$ is evaporation time. Please note that the reported photothermal evaporation rate should subtract the dark evaporation rate.

**Note S2 Energy flow mechanisms and thermal management strategies**

In the solar desalination process, solar energy is converted into heat, and the heat is transferred to the water-air interface to produce hot vapor through heat conduction of the material. The energy loss from the multi-level transfer of energy affects the overall evaporation efficiency. To achieve high thermal efficiency, heat loss due to conduction, convection and radiation should be minimized to maximize the effectiveness of heat utilization for steam generation. These heat conversion and transfer characteristics demonstrate the importance of energy management for evaporation system.

The energy flows are included in the solar-water conversion process: 1) the heat absorbed by evaporation of interfacial water, 2) heat transfer to the bulk seawater, 3) convective heat transfer with air and 4) thermal radiation to the environment. The energy flows of the system in equilibrium operation can be described as:

$$\begin{aligned} A_{proj}\alpha q_{solar}=C_{b}m\Delta T_{b}+A_{proj}h\left( T_{e}-T_{0} \right)+A_{proj}\kappa\frac{{\Delta T}_{e}}{{\Delta T}_{0}}+\epsilon\sigma A_{proj}\left( T_{e}^{4}-T_{0}^{4} \right)\#\left( S2 \right) \end{aligned}$$

where $q_{solar}$, $C_{b}$, $m$, $\Delta T_{b}$, $h$, $T_{e}$, $T_{0}$, $\kappa$, $\frac{{\Delta T}_{e}}{{\Delta T}_{0}}$, $\epsilon$, $\sigma$ are incident light density, specific heat capacity of bulk water, mass of bulk water, temperature change of bulk water, heat transfer coefficient of natural convection, evaporation interface temperature, ambient temperature (~27 °C), heat transfer coefficient of evaporator, temperature gradient between evaporator and ambient, thermal emissivity of evaporator and Stefan-Boltzmann constant (5.67 × 10^−8^ W m^−2^ K^−4^), respectively.

If no thermal or energy management is designed for the evaporator, the energy is estimated to be lost in radiation by 7%, convection by 5% and conduction by 43%. The main energy management strategy for solar desalination system is to reduce the heat transfer $\kappa$ from the evaporator, expanding the effective evaporation area allows more vapor to escape and then consume more enthalpy of phase transformation ($\Delta H$), adding insulation to the exterior of the evaporator to reduce the radiation heat transfer (reducing $T_{e}^{4}-T_{0}^{4}$). Therefore, it is necessary to apply energy storage materials in the evaporator to store the above heat losses, such as the energy storage hydrogel in this system, which will be able to contribute ~40% of waste heat utilization for the evaporator and is expected to increase the water production by ~20-25%.

Furthermore, we tested the temperature decay curves of the ESE and the evaporator without ESH after reaching maximum temperature. The results show that after 30 min of heat release, the temperature of the ESE decreased to 27.8°C, while that of the evaporator without ESH dropped to 22.1°C. The delayed cooling kinetics (0.72 vs 0.91 °C min^-1^) demonstrate active heat release from ESM crystallization during temperature decline, rather than passive environmental cooling. This sustained thermal energy delivery maintains the observed 0.53 kg m⁻² h⁻¹ dark evaporation rate (Fig. S14).

The ESE achieves thermal management through a synergistic mechanism combining PVA and ESMs (Fig. S15). During high-temperature phases (e.g., solar irradiation), the solid-phase PCM core (n-octadecane) within the microcapsules undergoes an endothermic phase change (solid→liquid), absorbing and storing thermal energy. During cooling phases (e.g., nighttime or reduced illumination), the liquid PCM core releases stored energy through exothermic crystallization (liquid→solid), maintaining interfacial evaporation. The hydrophilic PVA network ensures uniform thermal distribution by facilitating efficient heat transfer from the photothermal layer to the PCMs. Simultaneously, the hydrogel’s mechanical robustness maintains microcapsule integrity during repeated phase transitions. The system autonomously alternates between energy storage (daytime) and release (nighttime), mitigating thermal intermittency. This phase-change energy storage coupled with hydrogel-enabled thermal management dual mechanism ensures stable evaporation performance under variable environmental conditions.

**Note S3 Salt gradient transport and** **salt transport mechanism**

In the evaporator (ESE), the concentration gradient is created due to the higher rate of evaporation at the photothermal interface, where the salinity is higher than that of the bulk seawater. The high concentration of salt is diffusively transferred to the bulk seawater along the concentration gradient below the evaporation interface. The internal pore structure of the evaporator takes a decisive role in salt transfer, and this process determines the subsequent power generation efficiency. The transport of salt ions in the water is governed by the convection-diffusion equation:

$$\begin{aligned} \frac{\partial C}{\partial t}=D_{s,eff}\nabla^{2}c-\nabla\cdot\left( uc \right) \# \left( S3 \right) \end{aligned}$$

where$t$ represents time, $D_{s,eff}$ represents the effective mass diffusivity of salt ions in the evaporator, and $u$ represents the flow field. The $D_{s,eff}$ of an interfacial evaporator with a homogeneous porous structure can be reasonably estimated according to Eq. (S4):

$$\begin{aligned} D_{s,eff}=\mathcal{E}_{P}^{\frac{3}{2}}D_{S} \# \left( S4 \right) \end{aligned}$$

where $\mathcal{E}_{P}$ and $D_{S}$ represent the porosity of the evaporator and the intrinsic mass diffusivity of salt ions in water, respectively.

Since the ESE possessed a high porosity, it can increase $D_{s,eff}$, which promotes the extent of salt ion reflux along the salinity gradient. This high porosity structure provides 3 advantages: (1) rapid salt reflux due to high porosity eliminates surface salt deposition, (2) salt ions would move quickly along the salinity gradient into the bulk seawater, and (3) rapid salt reflux rapidly increases seawater salt concentration and promotes electrochemical reactions in bulk seawater.

This study investigated the salt transport mechanisms in seawater desalination through finite element simulations, with focused analysis on temperature fields and transport behaviors of water molecules and Na⁺. The model was developed within a coupled thermal-fluid-solute Multiphysics framework, where heat conduction was governed by the energy conservation equation and Fourier's law, while Na⁺ diffusion was modeled using Fick's law. Key parameters included: geometric dimensions (thickness 0.01 m, length 0.03 m), material properties (Na⁺ diffusion coefficient *D*_Na+_=5.9×10^-12^ m^2^ s^-1^, gas-liquid interface mass transfer coefficient is 1×10^-6^ m s⁻¹), ambient conditions (T_sur_ =293.15 K, 50% relative humidity), and energy inputs is *Q*_Sun_=1000 W m⁻², water latent heat of evaporation *h_w_*=44.9 kJ mol^-1^.

During solar-driven evaporation, the interfacial temperature reached approximately 323 K due to direct solar radiation absorption (Figs. S9a-b show the 3D and 2D *X-Z* plane temperature distributions of the hydrogel evaporator). This temperature increases induced interfacial water evaporation, transforming the originally uniform water content distribution in ESH into a vertical gradient from 96.4% (bottom) to 94.2% (top) (Fig. S9c), which drove bottom-to-top water migration (Fig. S9d), demonstrating the water replenishment capability to compensate for evaporation losses. Concurrently, interfacial evaporation increased Na⁺ concentration, establishing a reverse concentration gradient from 5.66% (top) to 3.5% (bottom) (Fig. S9e). Through the synergistic action of downward salt diffusion (Fig. S9f) and upward water transport, the interfacial salt concentration was maintained well below the ~26.4% safety threshold, effectively preventing salt saturation. These simulations results demonstrate that the hydrogel evaporator achieves highly efficient salt rejection through the tripartite synergistic mechanism of thermal-localized evaporation, directional water transport, and reverse salt diffusion.

**Note S4 Material selection for the evaporator and specific configuration and mechanism of the RED system**

PVA was selected as the hydrogel matrix due to its unique molecular structure and physicochemical properties that synergistically enhance the evaporator's performance. The presence of abundant hydroxyl groups along the PVA backbone enables superior hydrophilicity, facilitating rapid water transport through capillary action while maintaining structural stability in aqueous environments. These hydroxyl groups also form strong hydrogen bonds with water molecules, optimizing the water-vapor phase change efficiency at the evaporation interface. Additionally, PVA's flexible polymer chains and tunable crosslinking density allow for effective encapsulation of energy storage microcapsules (ESM) while maintaining mechanical robustness under repeated thermal cycling. The polymer's chemical compatibility with glutaraldehyde crosslinking further ensures stable integration of photothermal components and prevents phase separation during operation. PVA was also chosen for its optimal balance between water uptake capacity (critical for continuous evaporation) and thermal stability (essential for heat localization), while its cost-effectiveness and non-toxicity make it practical for scalable applications. These advantages collectively enable PVA to serve as an ideal matrix that simultaneously addresses the requirements of water transport, thermal management, and structural durability in the energy storage evaporator system.

The RED system consists of two rectangular half-cells and an ion-selective membrane. The rectangular half-cells are divided into seawater half-cell and river half-cell, each with dimensions of 10 cm × 5cm × 7cm, and the two half-cells are fastened in the center with a screw and rubber boot. The evaporator was placed in the seawater half-cell and illuminated by a xenon lamp (AM 1.5G) above. A condenser (20 cm×5 cm) was placed on top of the cell to facilitate the collection of clean water. The Ag/AgCl electrodes were placed into the corresponding half-cell, the seawater cell electrode was connected to the green wire and the river cell electrode was connected to the red wire of the electrochemical workstation. The diffusion of cations across the gradient led to charge separation, creating an electrochemical potential difference between the Ag/AgCl electrodes. This redox reaction between the two electrodes converted the internal ionic flow into an external electron flow, allowing efficient extraction of salt differential energy. The drainage design in WEC system features a 1 cm diameter pipeline connected to the bottom of the surface water half-cell in the RED device, with both inlet and outlet positioned on the surface water side to maintain optimal salinity gradients while ensuring efficient water circulation. This configuration prevents mixing between chambers, facilitates complete fluid exchange, and sustains stable operation.

In the high-salinity seawater, Na⁺ migrates through the Nafion membrane toward the low-salinity side, while Cl⁻ is blocked by the membrane, resulting in charge separation. At the anode, Ag reacts with Cl⁻ to form AgCl and releases electrons (Ag + Cl⁻ → AgCl + e⁻). At the cathode, AgCl captures electrons and is reduced to Ag, releasing Cl⁻ (AgCl + e⁻ → Ag + Cl⁻). The electrons released at the anode flow through the external circuit to the cathode, generating a continuous current. The entire process achieves sustained power generation through reversible electrode reactions and membrane selectivity, while evaporation-driven concentration maintains the salinity gradient.

The corresponding electrode reactions are:

$Ag+{Cl}^{-}\to AgCl+e^{-}$ (Seawater half-cell)

$AgCl+e^{-}\to Ag+{Cl}^{-}$ (River water half-cell)

The open circuit voltage (*Eoc*), short circuit current (*I_sc_*) and corresponding LSV curves of the RED system were examined using the electrochemical workstation (CHI600E).

**Note S5 Mechanisms of power generation in RED**

When liquids of different salt concentrations are separated by ion exchange membranes, the salt differential energy contained therein can be converted into external circuit electrical energy by transmembrane ion diffusion. This is a type of energy released by mixing solutions of different salt concentrations (Gibbs free energy, $\Delta G$), which can be calculated by:

$$\begin{aligned} \Delta G=2RTV_{1}c_{1}ln \frac{c_{1}\left( 1+\varphi\right)}{c_{1}+\varphi c_{2}}+2RTV_{1}{\varphi c}_{2}ln \frac{c_{2}\left( 1+\varphi\right)}{c_{1}+\varphi c_{2}}\#(S5)\# \end{aligned}$$

Where $R$ represents gas constant, $T$ represents temperature (K), $c_{1}$ and $c_{2}$ represent concentration in the high salt region and concentration in the low salt region, respectively, $\varphi$ represents the volume ratio of the low concentration solution to the high concentration solution, that is $\frac{V_{2}}{V_{1}}$.

Typically, salinity gradient power system consists of two solutions with different salinities separated by an ion exchange membrane composed of charged nanopores. Due to the salt concentration difference between both sides of the ion exchange membrane, ions can penetrate the nanopores of the membrane to diffuse from the high-concentration solution to the low-concentration solution. In case a cation exchange membrane is applied, the cations preferentially diffuse through the nano-pores in a directed manner, resulting in a net ion charge flow (difference between the cationic flux and the anionic flux). As a result, an electrical potential difference is generated across the membrane, typically referred to as osmotic electrostatic potential ($\Delta E_{osm}$). The osmotic electrostatic potential can be approximated by the Nernst equation:

$$\begin{aligned} \Delta E_{osm}=\frac{k_{B}T}{ze}Sln\frac{c_{1}}{c_{2}}\#\left( S6 \right) \end{aligned}$$

Where $\frac{k_{B}T}{ze}$ ≈ 5.7 mV (25 °C), and $S$ (ion selectivity) can be expressed as:

$$\begin{aligned} S=\frac{D_{+}-D_{-}}{D_{+}+D_{-}}\#\left( S7 \right) \end{aligned}$$

Where $D_{\pm}$ represents the effective diffusion constant of cations and anions.

In the process of extracting energy from the RED, the redox reaction at the electrode under the concentration gradient produces a potential drop ($E_{r}$) and the cation-selective membrane contributes only to the diffusion potential ($E_{d}$). In NaCl electrolyte:

$$\begin{aligned} E_{r}=\frac{RT}{F}ln \left( \frac{c_{1}y_{1}}{c_{2}y_{2}} \right)\# \left( S8 \right) \end{aligned}$$

$$\begin{aligned} E_{d}=\left( 2t_{+}-1 \right)\frac{RT}{F}ln \left( \frac{c_{1}y_{1}}{c_{2}y_{2}} \right)\# \left( S9 \right) \end{aligned}$$

where $R$, $T$, $F$, $c_{1}$, $c_{2}$, $y$ and $t_{+}$ represent gas constant, temperature (K), Faraday constant, concentration in the high salt region, concentration in the low salt region, the ionic activity coefficient corresponding to the concentration and number of ions migrating. $t_{+}=1$ and $t_{+}=0$ denote complete cation selectivity and complete anion selectivity, respectively.

The voltage detected by the electrochemical workstation is the sum of $E_{r}$ and $E_{d}$, so the apparent voltage ($E_{a}$) can be expressed as:

$$\begin{aligned} E_{a}=E_{r}+E_{d}=2t_{+}\frac{RT}{F}ln \left( \frac{c_{1}y_{1}}{c_{2}y_{2}} \right)\# \left( S10 \right) \end{aligned}$$

Similarly, in a RED-based electrochemical system, since it is mainly Na^+^ that is transported across the Nafion membrane in a concentration gradient, the current ($I$) resulting from the diffusion process can be calculated via the following equation:

$$\begin{aligned} I=\frac{AF^{2}}{RT}D_{NaCl}c_{1}\frac{\partial E_{a}}{\partial x}+FAD_{NaCl}\frac{\partial c}{\partial x} \# \left( S11 \right) \end{aligned}$$

Where $A$ is the diffusion cross area, $D_{NaCl}$ is ion diffusion coefficient, $\frac{\partial c}{\partial x}$ is the concentration gradient.

The maximum output power $P_{max}$ of the RED can be calculated from the voltage-current curve:

$$\begin{aligned} P_{max}=\frac{E_{OC}I_{SC}}{4}\#(S12)\# \end{aligned}$$

where $E_{OC}$ and $I_{SC}$ represent the open-circuit voltage and short-circuit current, respectively.

**Note S6 Carbon offsets provided by the operation of the WEC system**

In contrast to conventional seawater desalination systems that rely on fossil fuels or electricity, the WEC system operates without the need for additional chemicals or external energy inputs, thereby achieving a zero-greenhouse gas (GHG) emission process. To quantify the carbon offset benefits of the WEC system during its operation, the Emission Factor (EF) methodology was employed to comprehensively assess the integrated process of solar-driven seawater desalination, electricity generation, and irrigation water provision. The evaluation system boundary focuses on the operation process of the WEC system, covering the comprehensive processes of freshwater, electricity, and irrigation water production driven by it, without considering the losses generated during the supply process. To ensure a robust estimation of the system’s greenhouse gas mitigation potential, specific operational scenarios were meticulously designed. The scenario involves the continuous operation of a 1000 m^3^ WEC system in Haikou, China, aimed at producing fresh water, electricity, and irrigation water throughout the year. Within this context, the annual output of the WEC system, encompassing all three products, is multiplied by the EF to precisely calculate the annual carbon offset generated by the system.

In this study, the quantitative analysis was conducted based on the following premise assumptions:

(1) System operation stability assumption:

It is assumed that the WEC system operates under steady-state conditions within the evaluation period (1 year). The impacts of equipment aging, fault maintenance, or shutdowns caused by extreme weather are not considered for the time being. Only the continuous operation performance under the design conditions is focused on.

(2) Yield benchmark assumption:

The yield parameters of fresh water, electricity, and irrigation water are based on the monitoring data of the WEC system during the laboratory operation stage. The values are the statistical means under the ideal conditions without external intervention, and the short - term yield deviations caused by fluctuations in environmental parameters during actual operation are ignored.

(3) Sunshine hours correlation assumption:

It is assumed that the total output of the system (fresh water, electricity, and irrigation water) has a linear positive - proportional relationship with the effective sunshine hours. This relationship is limited to the geographical area and climatic conditions set in the evaluation scenario, and the non - linear impacts of special weather such as cloudy and rainy days are not included.

(4) External interference exclusion assumption:

During the evaluation process, only the technical characteristics of the WEC system itself are focused on. Interferences from non - system inherent factors such as human operation adjustments, production scheduling changes, and policy interventions on the operation efficiency are excluded to isolate and analyze the carbon offset potential at the pure technical level.

The monthly average daily sunshine duration across the selected region was meticulously analyzed using historical meteorological data sourced from the China Meteorological Administration (CMA), as depicted in Figs. S24-25. This dataset was employed to compute the diurnal and nocturnal operational periods of the WEC system, subsequently enabling the quantification of freshwater, electricity, and irrigation water yields. Carbon emissions arising from freshwater production in conventional drinking water treatment facilities were systematically aggregated from multiple scholarly investigations, serving as the foundation to derive the greenhouse gas offset EF for the seawater desalination process. Recognizing the heterogeneity in the unit conventions of greenhouse gas measurement across distinct studies, a normalization protocol was implemented to harmonize the raw data into a unified EF, as summarized in Table S2. The EF for carbon offset associated with freshwater production by the WEC system was then defined as the mean of the probability distribution of these normalized emissions factors. Furthermore, in alignment with the potential deployment regions of the WEC system, a statistical synthesis of the average EF for electricity generation was conducted across several low- to mid-latitude countries in Asia, detailed in Table S3. The GHG offset attributable to the system’s electricity generation was thereby calculated based on the aggregated average EFs of these nations. It is noteworthy that, although the irrigation water generated by the system lacks measurable carbon offset potential, it introduces novel opportunities for enhancing local agricultural productivity while inherently avoiding greenhouse gas emissions.

The selected region for this assessment utilizes the Surface Solar Radiation (SSR) dataset contains nearly 36-year (1983.7-2018.12) high-resolution (3 h, 10 km) global SSR (surface solar radiation) dataset [S1], which can be used for hydrological modeling, land surface modeling and engineering application. The dataset was produced based on ISCCP-HXG cloud products, ERA5 reanalysis data, and MODIS aerosol and albedo products with an improved physical parameterization scheme. This SSR dataset will contribute to the land-surface process simulations and the photovoltaic applications in the future. The unit is W m^-2^, instantaneous value. Based on the global latitudinal solar radiation distribution data, the light intensity in mid-to-low latitude regions of Asia is relatively stable (Figs. 26-S29).

To ensure that the carbon offsets assessment more accurately reflects real-world conditions, we have chosen Hainan Province in China as the specific evaluation area. This selection is based on the Hainan Provincial Government’s comprehensive strategy detailed in the Hainan Carbon Peak Implementation Plan [S2], which aims to transform Hainan into a “low-carbon island” and explore innovative models of zero-carbon energy utilization tailored for island environments. The strategy specifically emphasizes “zero-carbon, smart, and circular” development, targeting a clean energy installed capacity ratio of 85%, achieving a farmland irrigation water utilization coefficient of 0.58, and expediting the construction of the “Hainan Clean Energy Island.” Consequently, this assessment identifies Haikou City in Hainan Province as the appropriate region for carbon offsets accounting, specifically to evaluate the carbon reduction potential arising from the application of the WEC system in this area.

**Note S7** **Energy flow diagram and energy utilization efficiency**

The input sunlight intensity is 1000 W m^−2^, and the energy is converted into three main energy streams: (1) the energy for water evaporation, (2) the reflection energy loss, (3) other ways of heat loss (like heat convection and radiation). Thus, the energy utilization of solar for water evaporation equation:

$$\begin{aligned} \eta=\frac{h_{v}\times WER}{P_{Solar}}\#\left( S13 \right) \end{aligned}$$

Where, $h_{v}$ is the enthalpy of evaporation of ESE, $WER$ is the evaporation rate, and $P_{Solar}$ is the intensity of light. As a result, the energy utilization of solar for water evaporation is approximately 93.1%.

The Gibbs free energy ($\Delta G$) resulting from mixing liquids of different concentrations can be calculated by Equation 4. For the ΔG, energy conversion is performed by the reverse electrodialysis (RED) technique. Theoretically, the energy per litre that becomes available when high-salinity seawater (>0.6 mol L^−1^) is mixed with surface water (~0.01 mol L^−1^) is larger than when simple seawater (0.6 mol L^−1^) is mixed with surface water (~0.01 mol L^−1^, ~2 kJ). Therefore, the use of high-salinity seawater is expected to greatly improve the salinity-gradient energy generated by the solar desalination process. According to experiments, the corresponding power density for mixing seawater and surface water is ~0.11 W m^-2^, which is increased to ~0.30 W m^-2^ in the ESE-based desalination process, resulting in a power density ~3 times higher and an increase in energy extraction efficiency of 173%. In addition, system drainage is used for cultivation during the system process, enabling a seamless integration of the desalination-power generation-cultivation cycle. The energy flow diagram of WEC system is shown in Fig. S30.

**Note S8** **Performance of the WEC system in real outdoor conditions**

To investigate the practical operation of the WEC system, an outdoor testing system was constructed based on ESE (Fig. 32a). A full-day outdoor experiment was conducted on May 22, 2025. This testing enabled performance evaluation throughout complete diurnal cycles while accounting for solar intensity variations and environmental fluctuations. The tests were performed in Harbin, Northeast China, with outdoor temperature and humidity shown in Fig. 32b. The maximum daytime temperature reached ~20.8°C, while the minimum nighttime temperature was ~10 °C, with relative humidity ranging between 30%-70%. A photometer recorded solar irradiance at different times (Fig. 32c), with a peak value of 0.843 kW m^-2^. Under daytime conditions, the mass change of WEC was ~8.5 kg m⁻², and the power output reached ~0.26 W m^-2^. Under non-light conditions the power output was ~0.11 W m^-2^ (Fig. 32d). The outdoor operational results demonstrate that the WEC system possessed excellent operational feasibility, which enhances the practicality of implementing efficient seawater desalination and power generation.

**Table S1** Comparison of ESE and system integration performance in this paper with other published literature

| **Materials** | **Evaporation rate (kg m^-2^ h^-1^)** | **Duration of operation** | **Additional functions** | **Refs.** |
| --- | --- | --- | --- | --- |
| Carbon black, cotton fabric | 1.37 | 5 d | / | [S3] |
| PPG, PDMS, PU, CNTs | 1.34 | 10 d | Self-healing | [S4] |
| CNTs, fiber mats | 1.94 | / | / | [S5] |
| Melamine foam, graphite | 1.38 | ~0.3 d | / | [S6] |
| PVDF porous membrane | 1.58 | ~0.5 d | / | [S7] |
| Carbon black, PMMA | 1.30 | 16 d | / | [S8] |
| Porous paper, polypyrrole | 1.88 | 1 d | / | [S9] |
| Wood | 1.79 | / | / | [S10] |
| Carbon black,  CuO | 1.88 | / | Antibacterial | [S11] |
| Carbonized wood | 1.04 | ~4 d | / | [S12] |
| Wood | 1.35 | 10 d | / | [S13] |
| **PVA, PDMS, bio-graphene** | **1.91** | **21 d** | **Water production**  **electricity generation**  **crop irrigation**  **thermal management** | **This work** |

**Table S2** Statistics on greenhouse gas EF of freshwater production

| Num. | EF (kgCO_2_e m^-3^) | Refs. |
| --- | --- | --- |
| 1 | 0.0968 | [S14] |
| 2 | 0.0830 |  |
| 3 | 0.0323 |  |
| 4 | 0.04149 |  |
| 5 | 0.0573 | [S15] |
| 6 | 0.0626 |  |
| 7 | 0.0565 |  |
| 8 | 0.08023 |  |
| 9 | 0.0891 |  |
| 10 | 0.0975 |  |
| 11 | 0.1028 |  |
| 12 | 0.108 |  |
| 13 | 0.1240 |  |
| 14 | 0.1513 |  |
| 15 | 0.1932 |  |
| 16 | 0.2082 |  |
| 17 | 0.2338 |  |
| 18 | 0.2373 |  |
| 19 | 0.26112 |  |
| 20 | 0.09 | [S16] |
| 21 | 0.46 | [S17] |

**Table S3** Greenhouse gas EF of electricity production in low- to mid-latitude countries in Asia

| Country | GHG Intensity (kgCO_2_e kWh^-1^) |
| --- | --- |
| China | 0.5942 |
| Japan | 0.461 |
| South Korea | 0.4113 |
| India | 0.7132 |
| Indonesia | 0.7848 |
| Singapore | 0.3449 |

The data are derived from public disclosures by the official authorities of the countries.


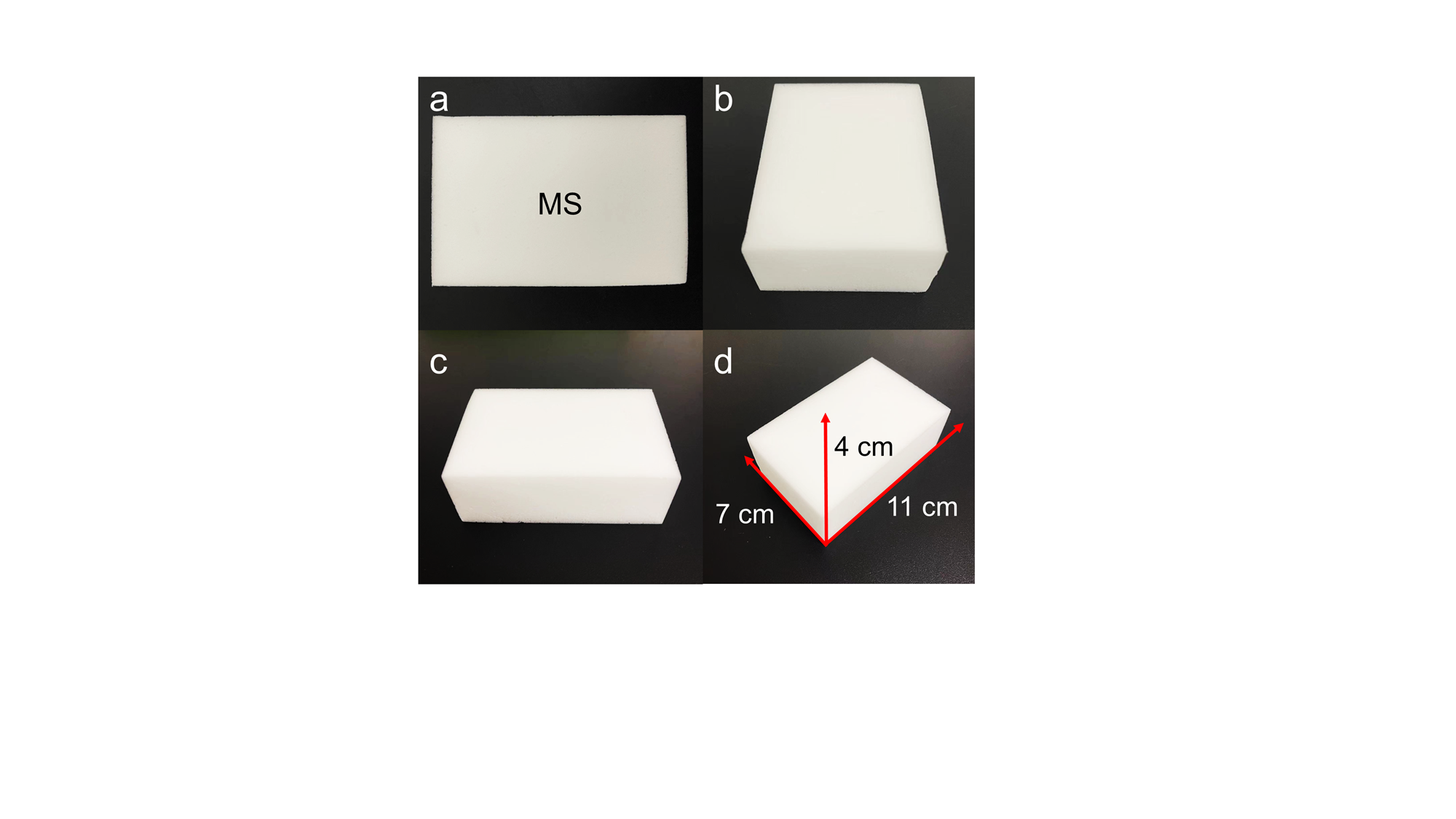


**Fig. S1** Optical photograph of the MS used (11 cm × 7 cm × 4 cm, surface area 77 cm^2^)


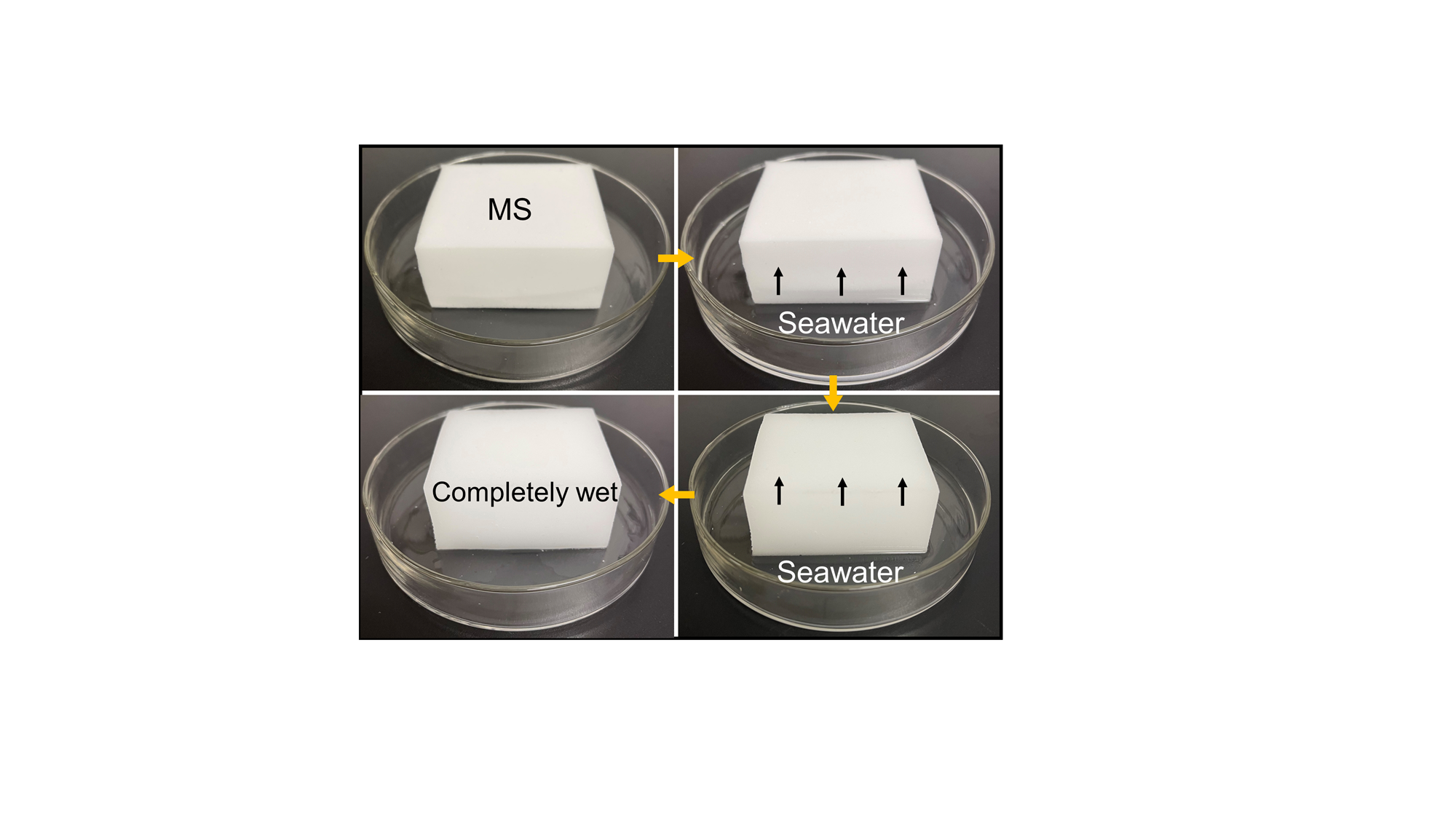


**Fig**. **S2** The excellent hydrophilicity of MS leads to its rapid wetting by seawater


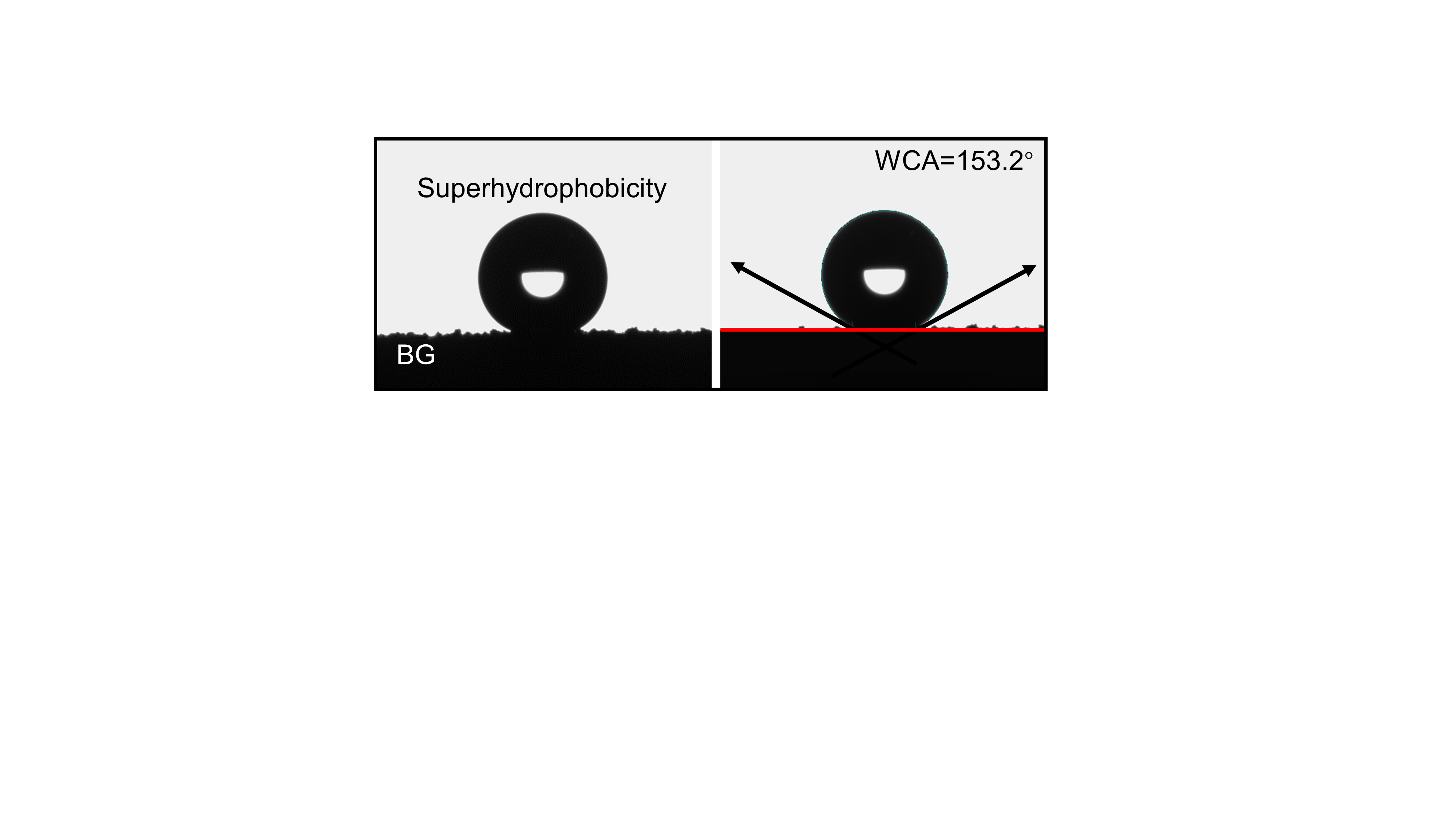


**Fig. S3** The hydrophobic BG surface exhibited superhydrophobicity with WCA = 153.2°


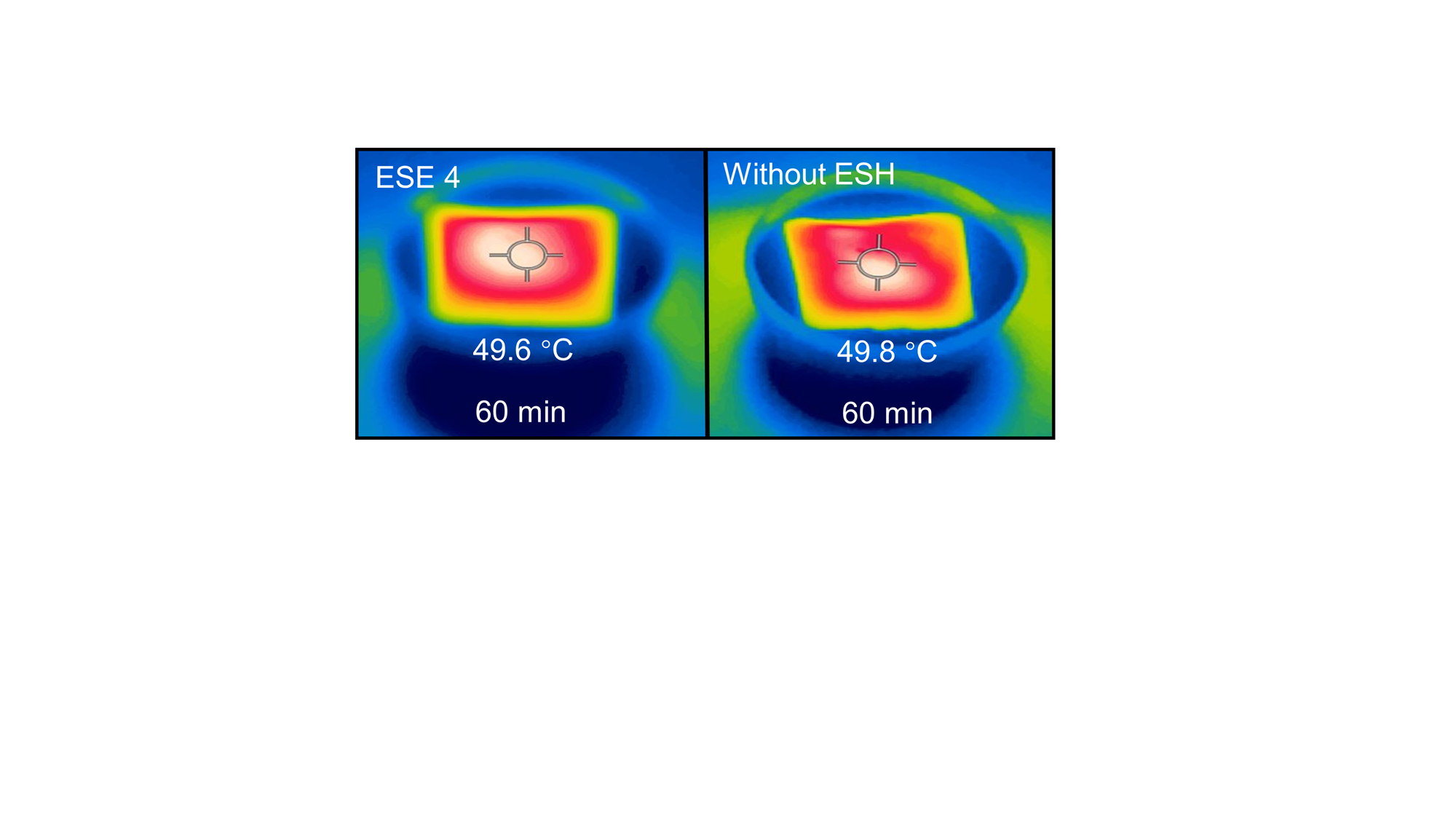


**Fig.** **S4** The surface temperatures were 49.6°C and 49.8°C for ESE 4 and without ESH, respectively, indicating that the addition of ESH did not affect the surface temperature of ESE 4


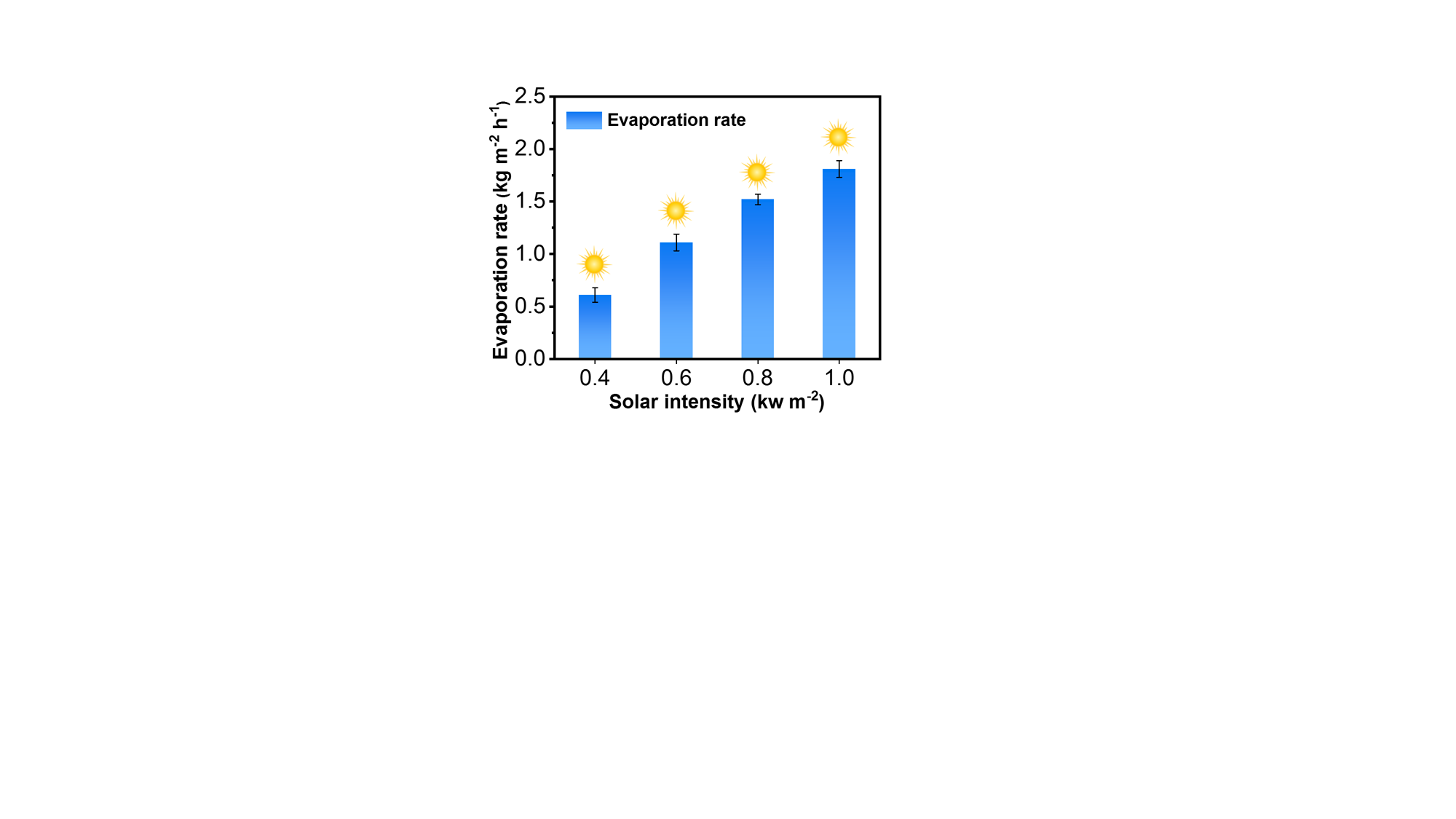


**Fig.** **S5** Evaporation rates of ESE at different solar radiation intensities


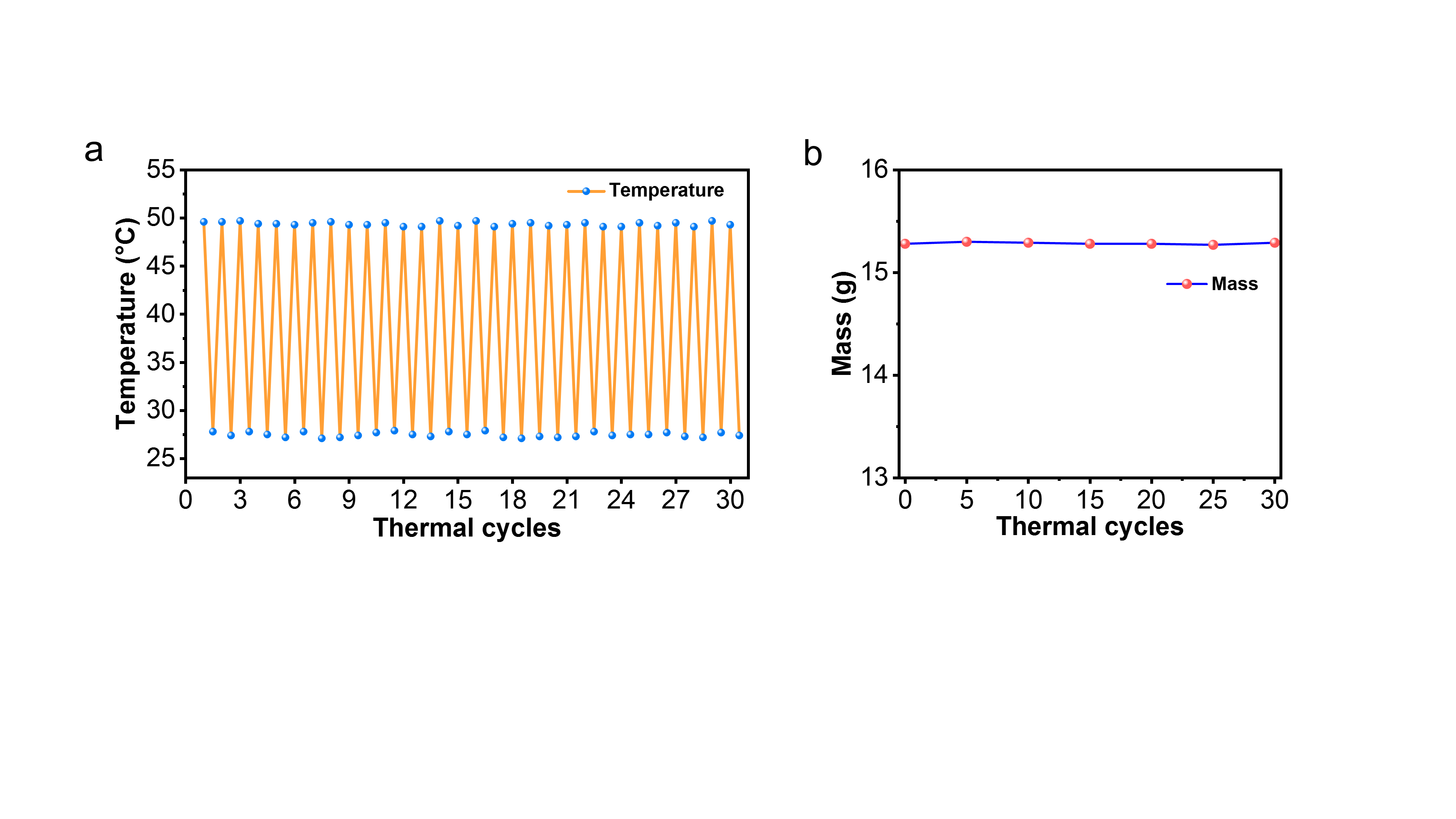


**Fig. S6 a)** Thermal cycle stability and **b**) mass change of ESE after 30 thermal cycles


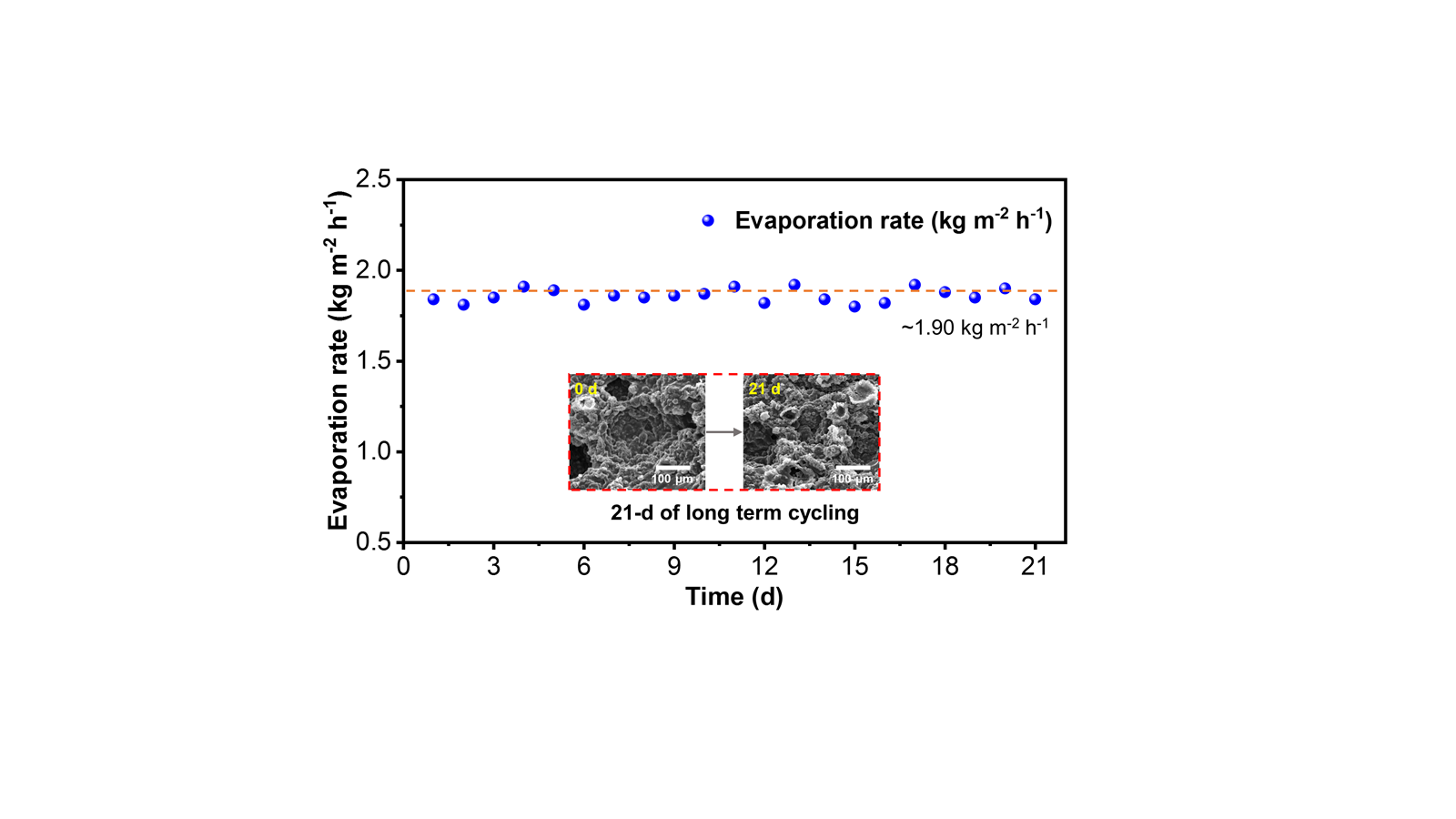


**Fig. S7** Evaporation rates distribution of the ESE under 21 d of continuous operation (inset shows SEM images of the microcapsule/hydrogel composite at 0 d and 21 d)


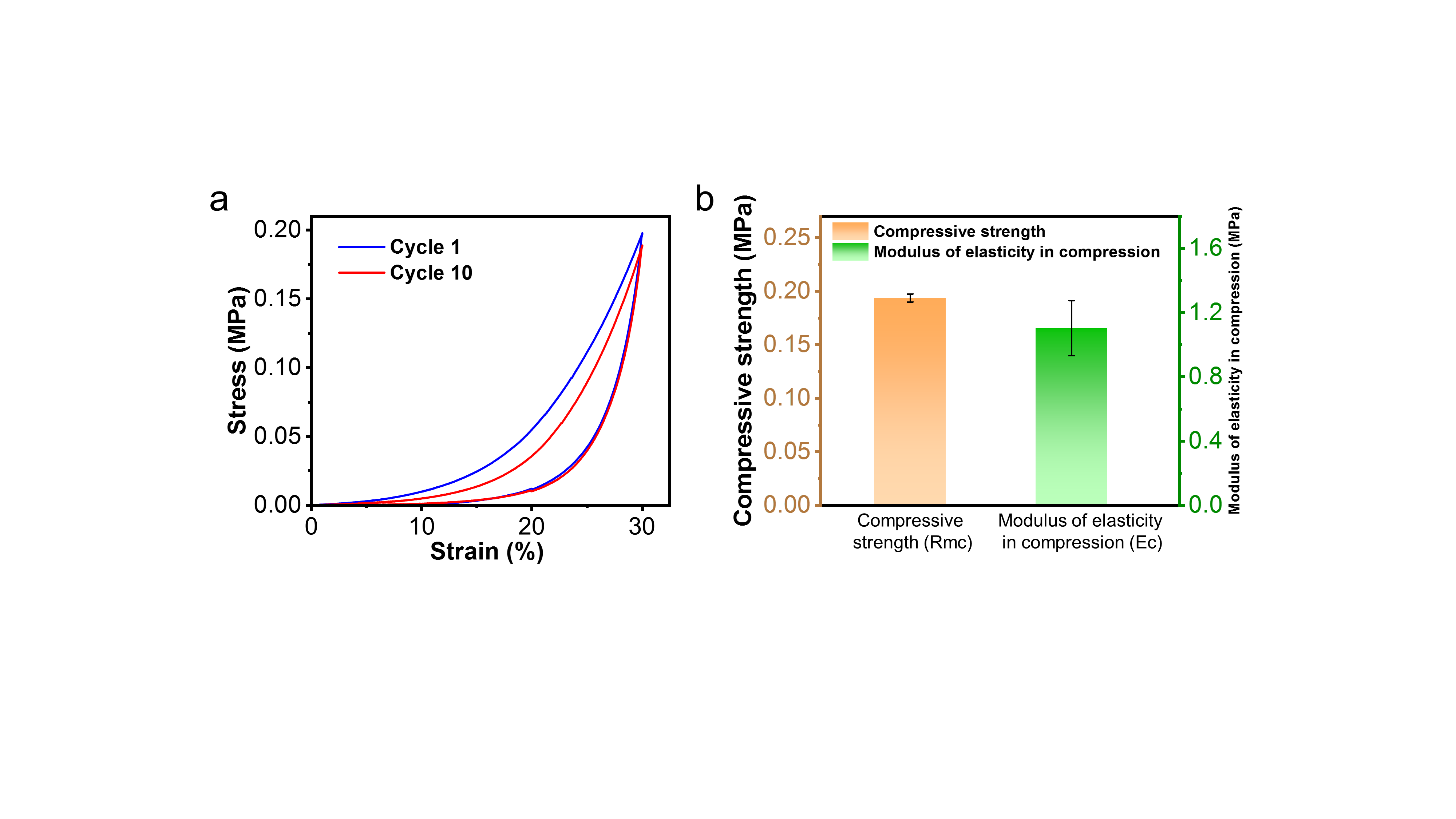


**Fig.** **S8** **a**) Stress-strain curve of ESE. **b**) Compressive strength (*Rmc*) and modulus of elasticity in compression (*Ec*) of ESE

**
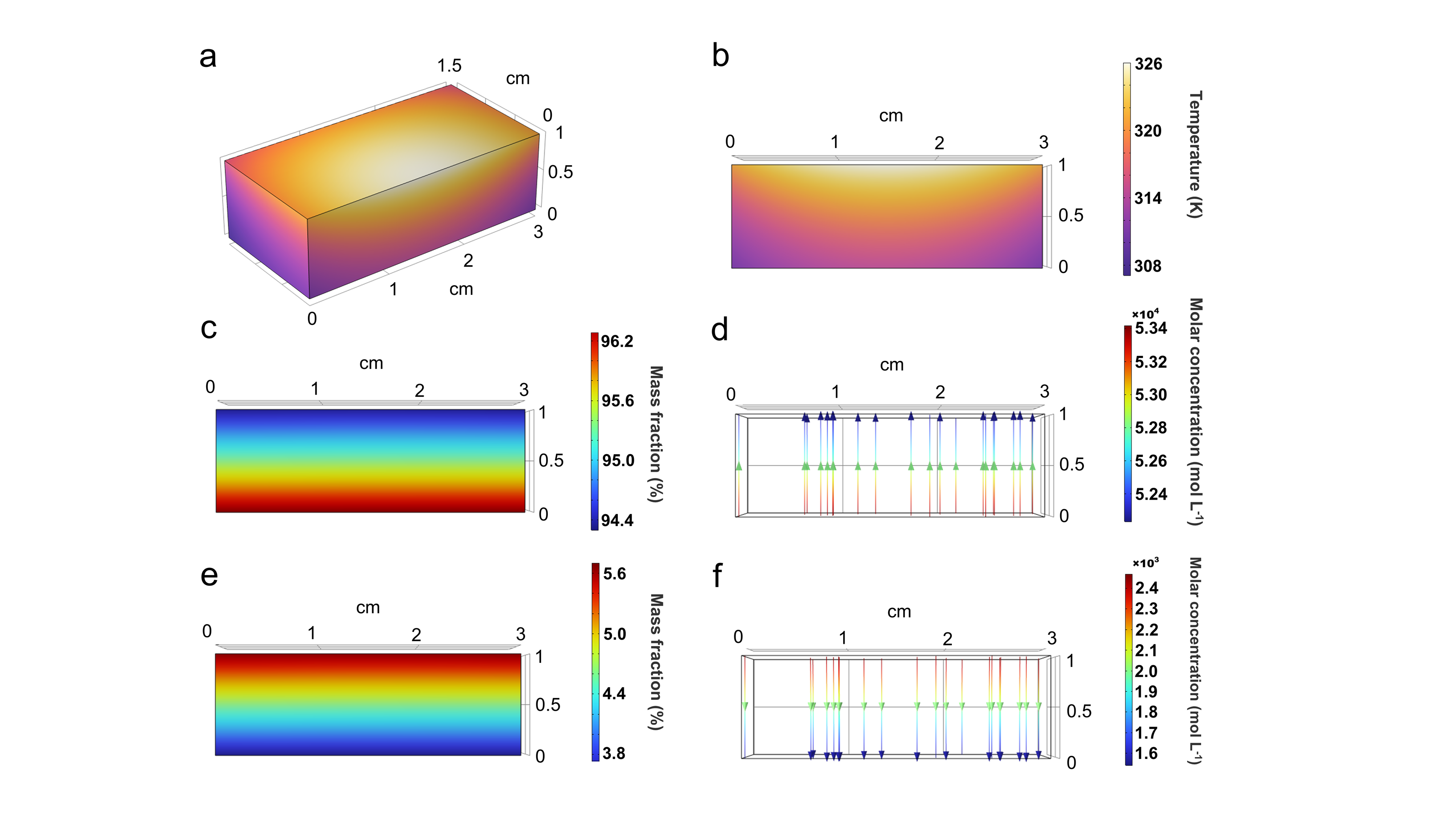
**

**Fig.** **S9** **a**) Three-dimensional temperature field distribution during evaporation process. **b**) Two-dimensional temperature field distribution in the *X-Z* plane. **c**) Mass fraction distribution of H_2_O in ESH. **d**) Molar concentration distribution of H_2_O in ESH. **e**) Mass fraction distribution of Na⁺ in ESH. **f**) Molar concentration distribution of Na⁺ in ESH


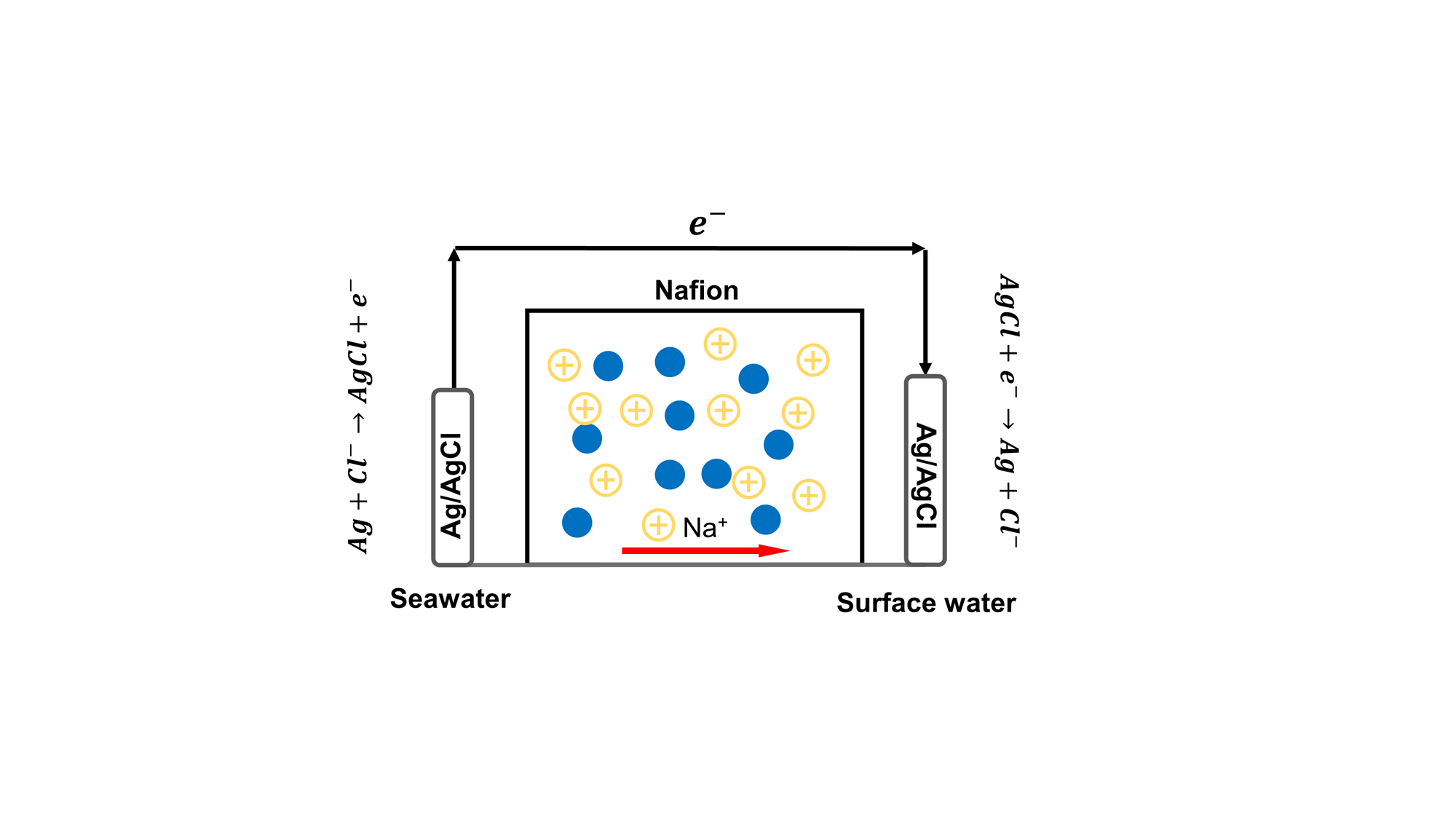


**Fig.** **S10** The generation mechanism of desalination-based RED system


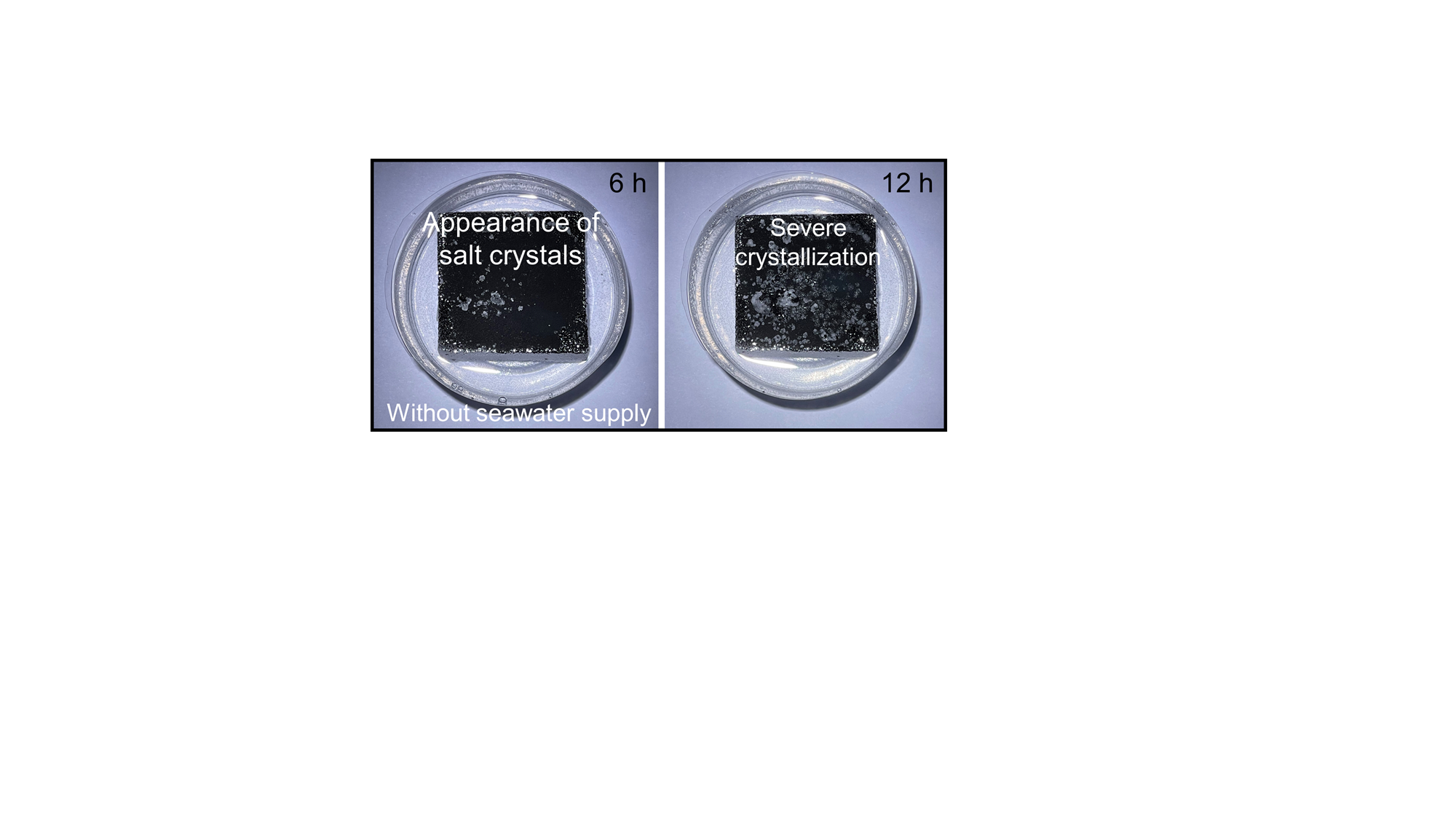


**Fig.** **S11** The evaporation process without seawater supply resulted in saturation of seawater and precipitation on the evaporator surface, which seriously affected the evaporation process


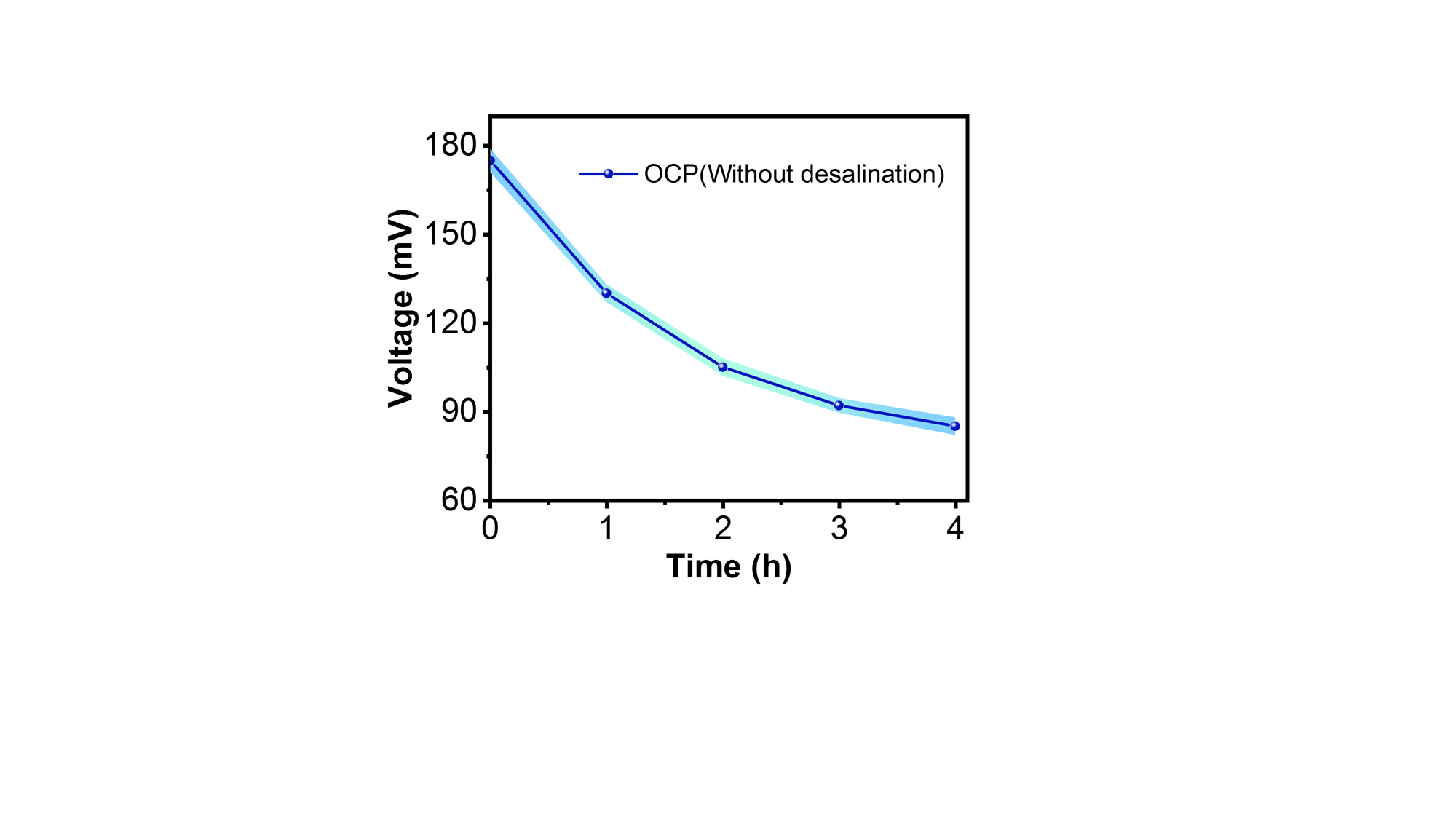


**Fig.** **S12** The curve of OCP for RED without desalination. The OCP decreased from ~175 mV to ~85 mV after 4 h of operation


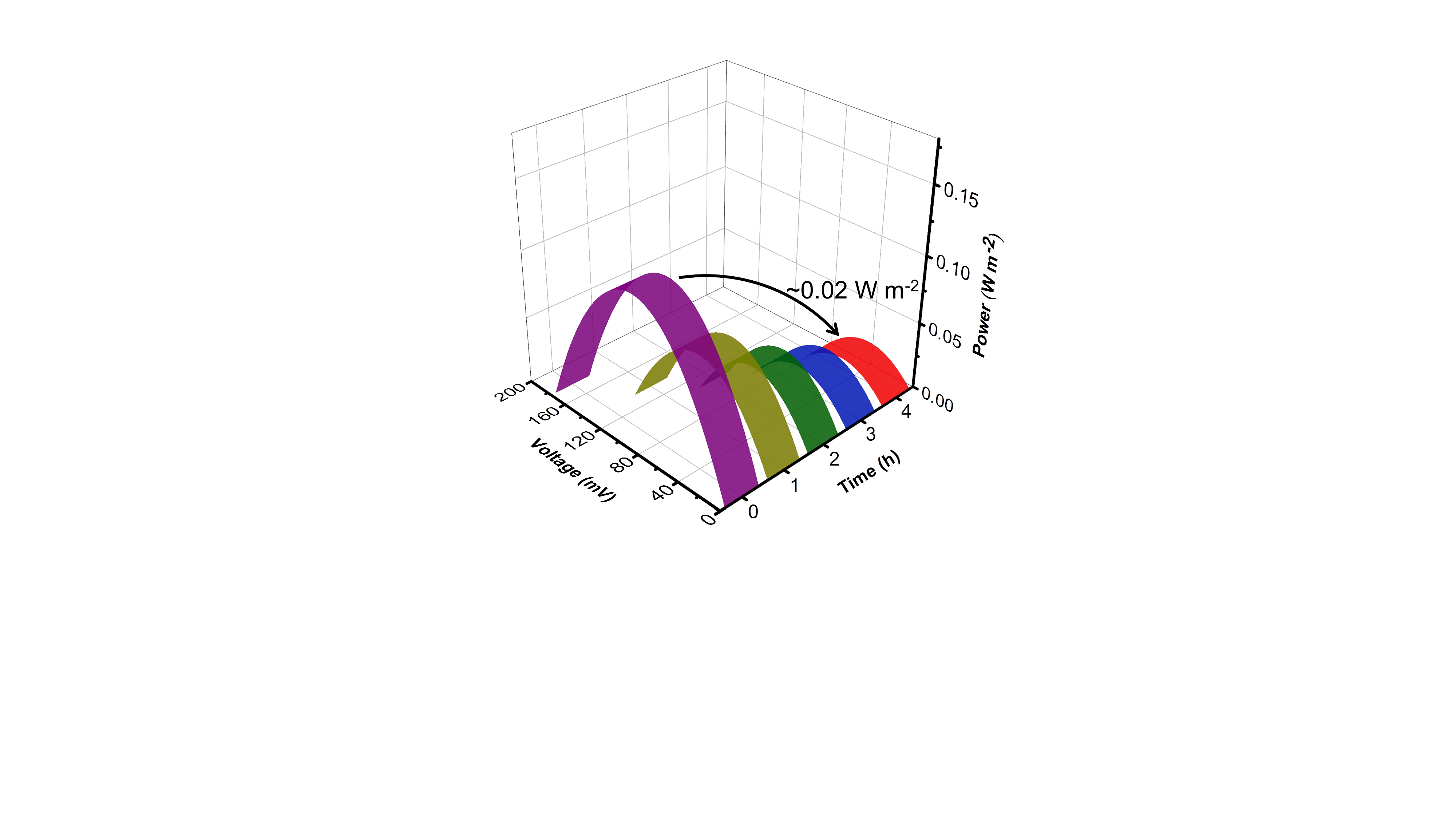


**Fig.** **S13** Dynamics of *P_max_* during operation to steady state (1-4 h) for RED without desalination


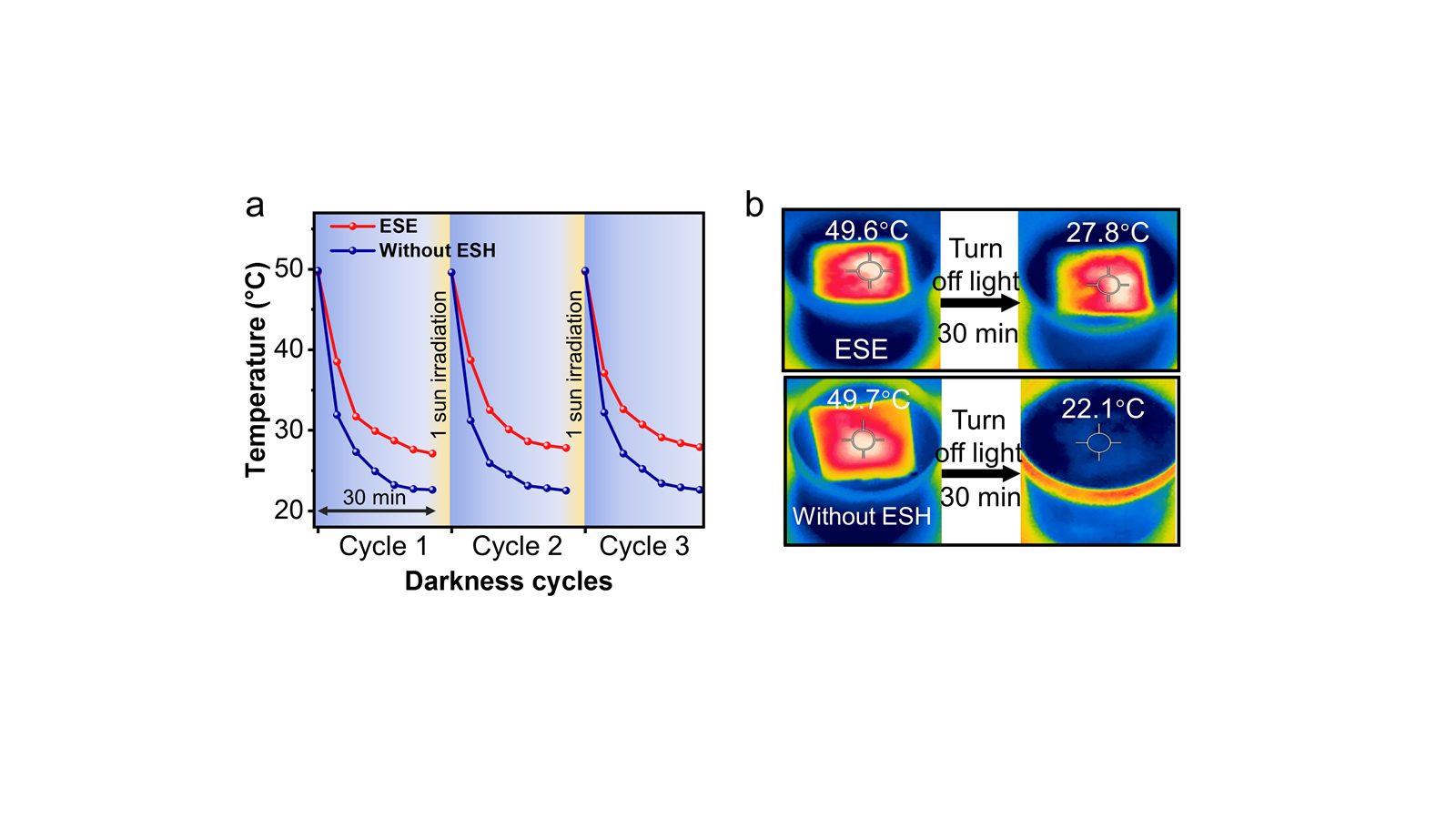


**Fig.** **S14** **a**) temperature decay curves of the ESE and the evaporator without ESH. **b**) Infrared images of the cooling process of ESE and evaporator without ESH


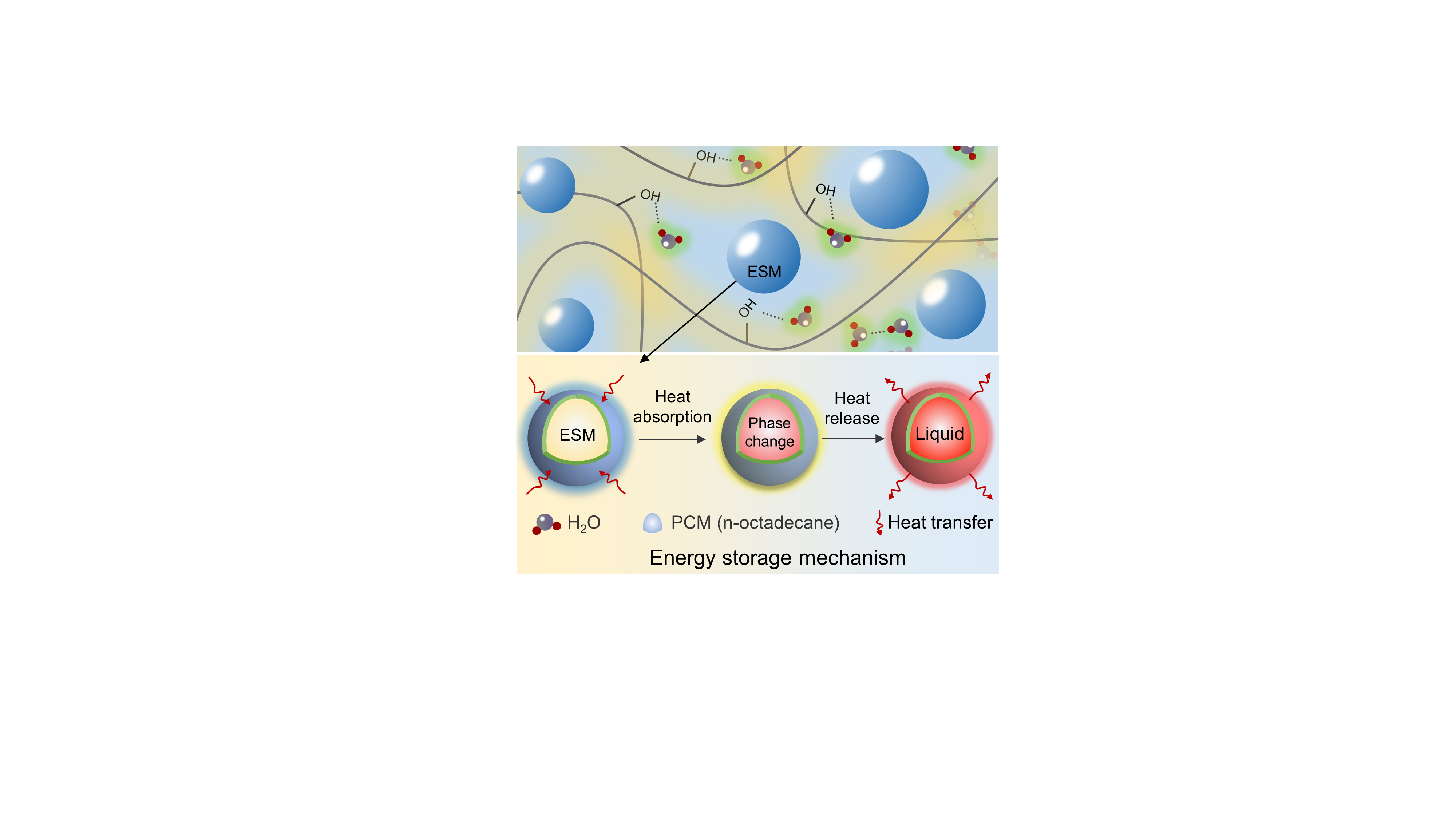


**Fig.** **S15** The mechanism of thermal management function through synergy between PVA and ESMs


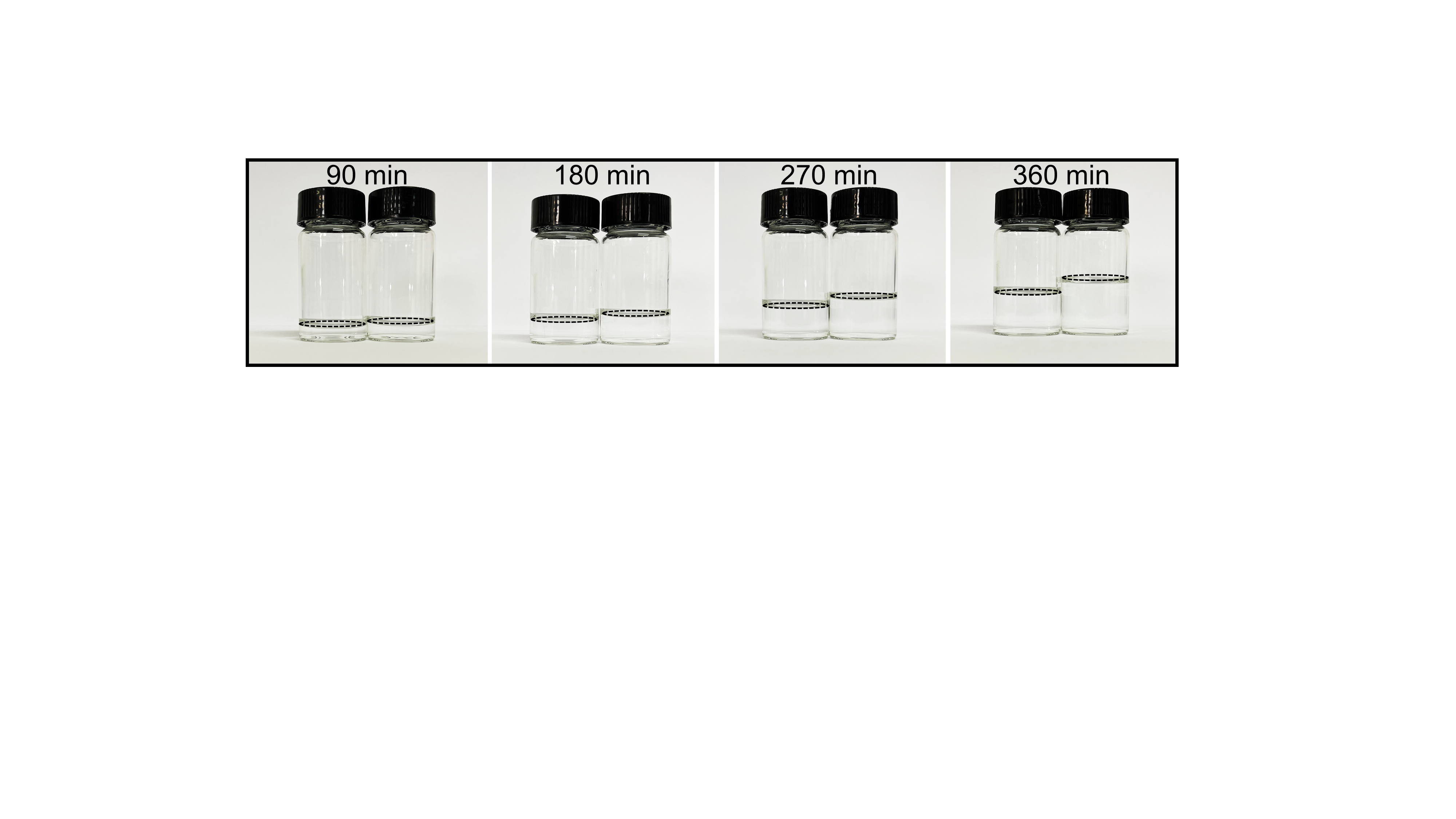
**Fig.** **S16** Photographs of collected water by turning on-off light in 4 cycles under 1 sun


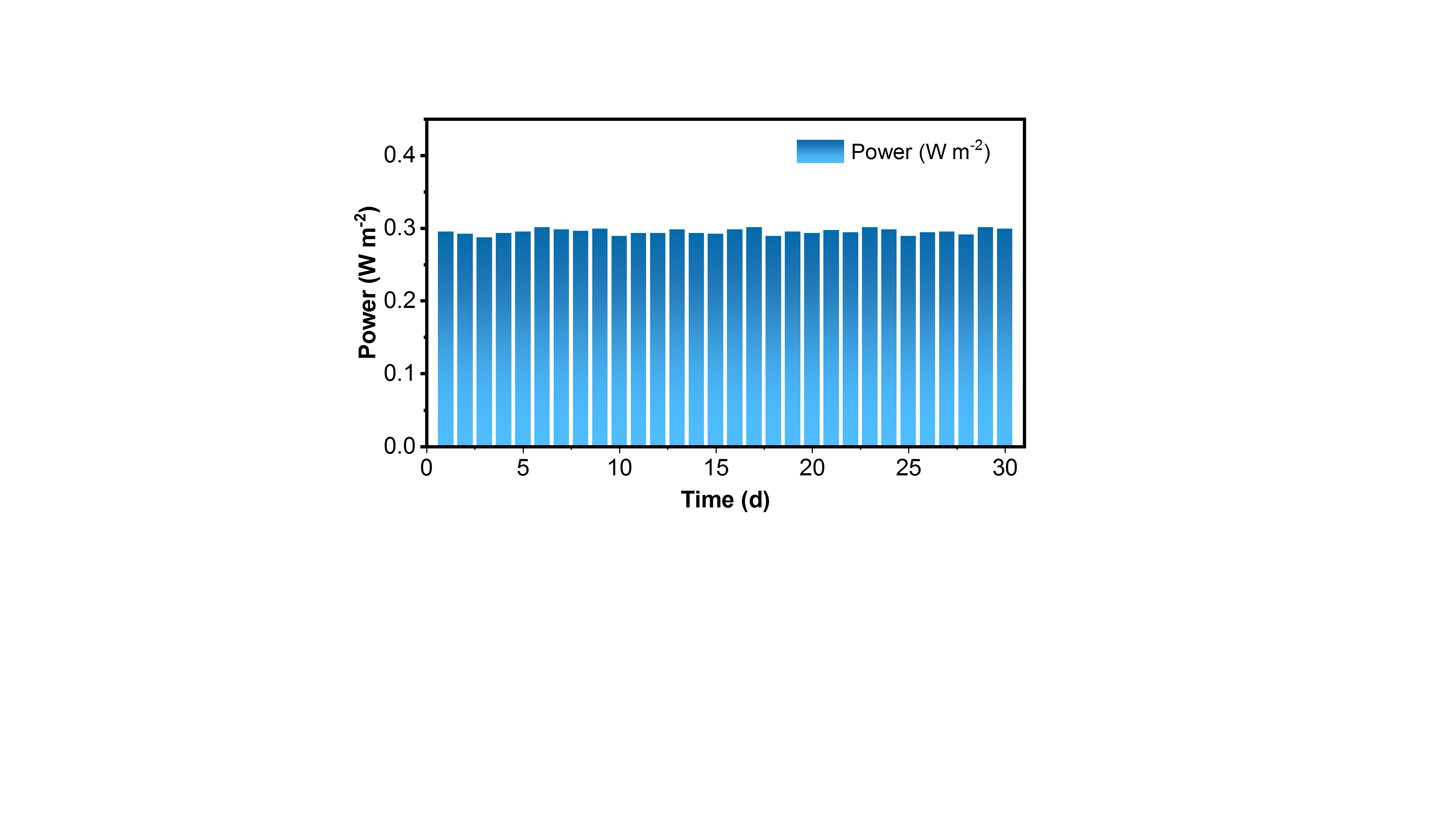


**Fig.** **S17** The power output of the 30-d cycle operation showed that *P_max_* were maintained at a steady state of ~0.3 W m^-2^


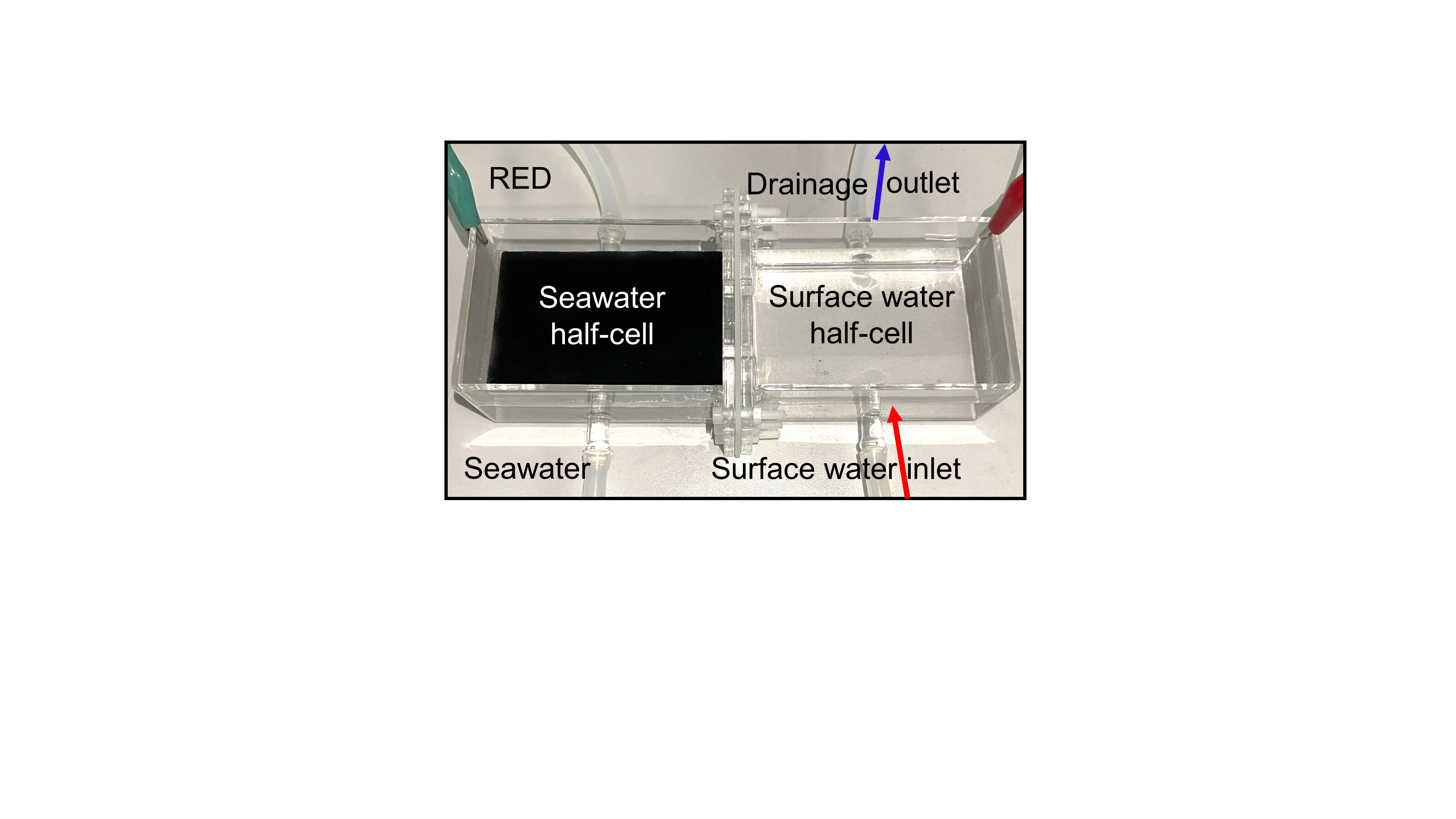


**Fig. S18** The details of the drainage design in the WEC system


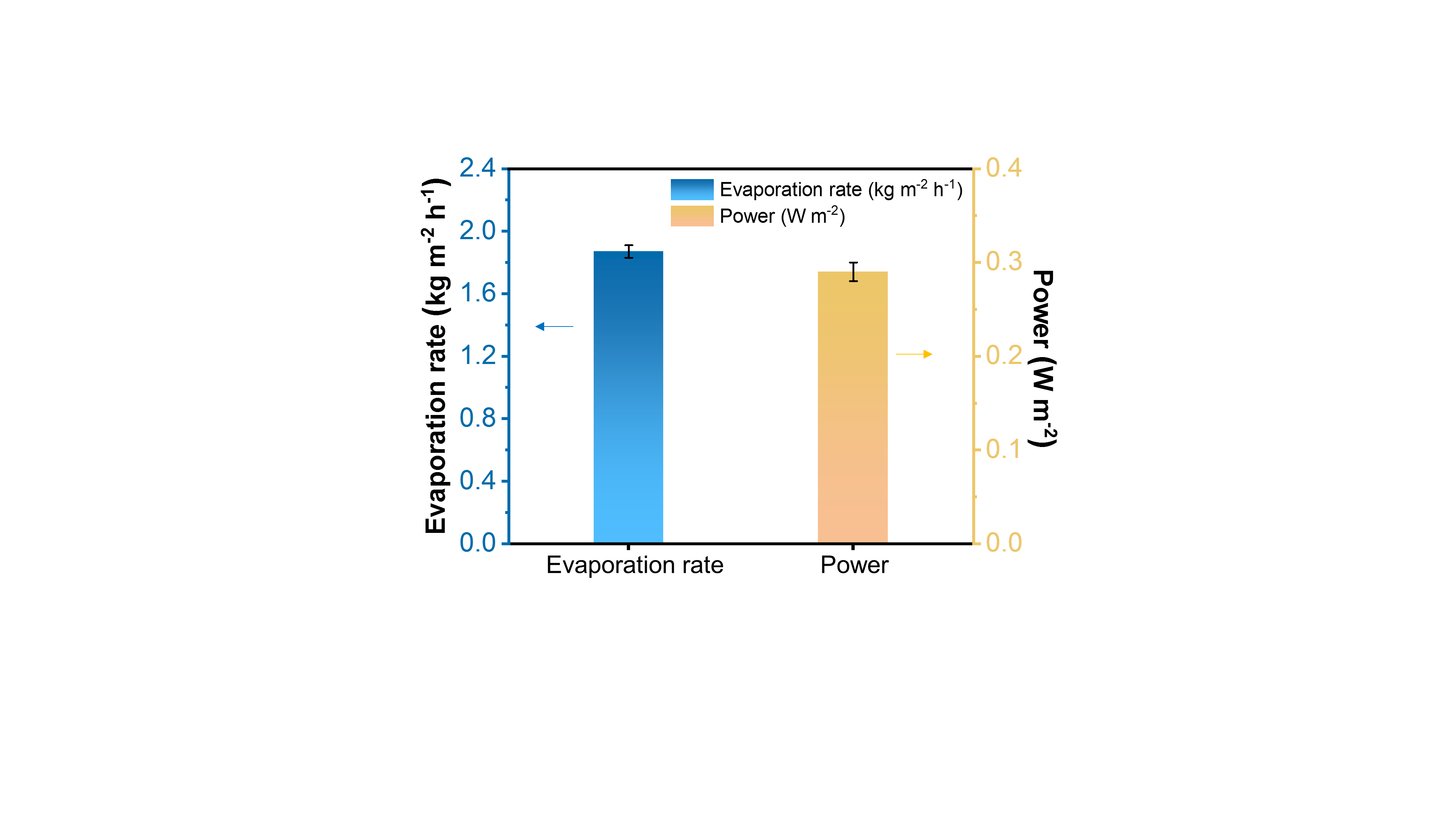


**Fig.** **S19** After the system was running at a steady state, the evaporation rate and *P_max_* were ~1.91 kg m^-2^ h^-1^ and ~0.29 W m^-2^, respectively


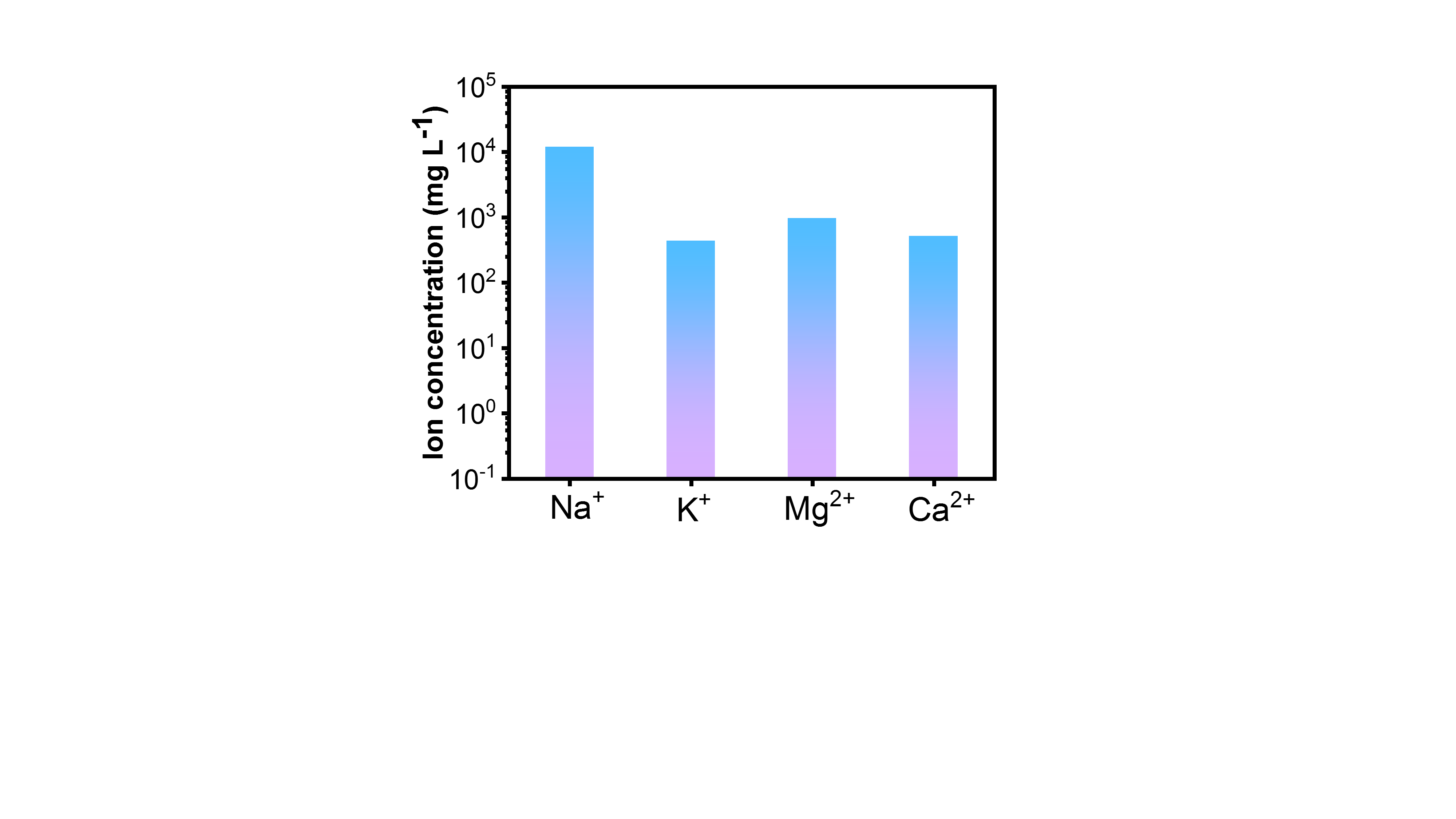


**Fig.** **S20** Concentration of seawater offshore China (Bohai Sea)


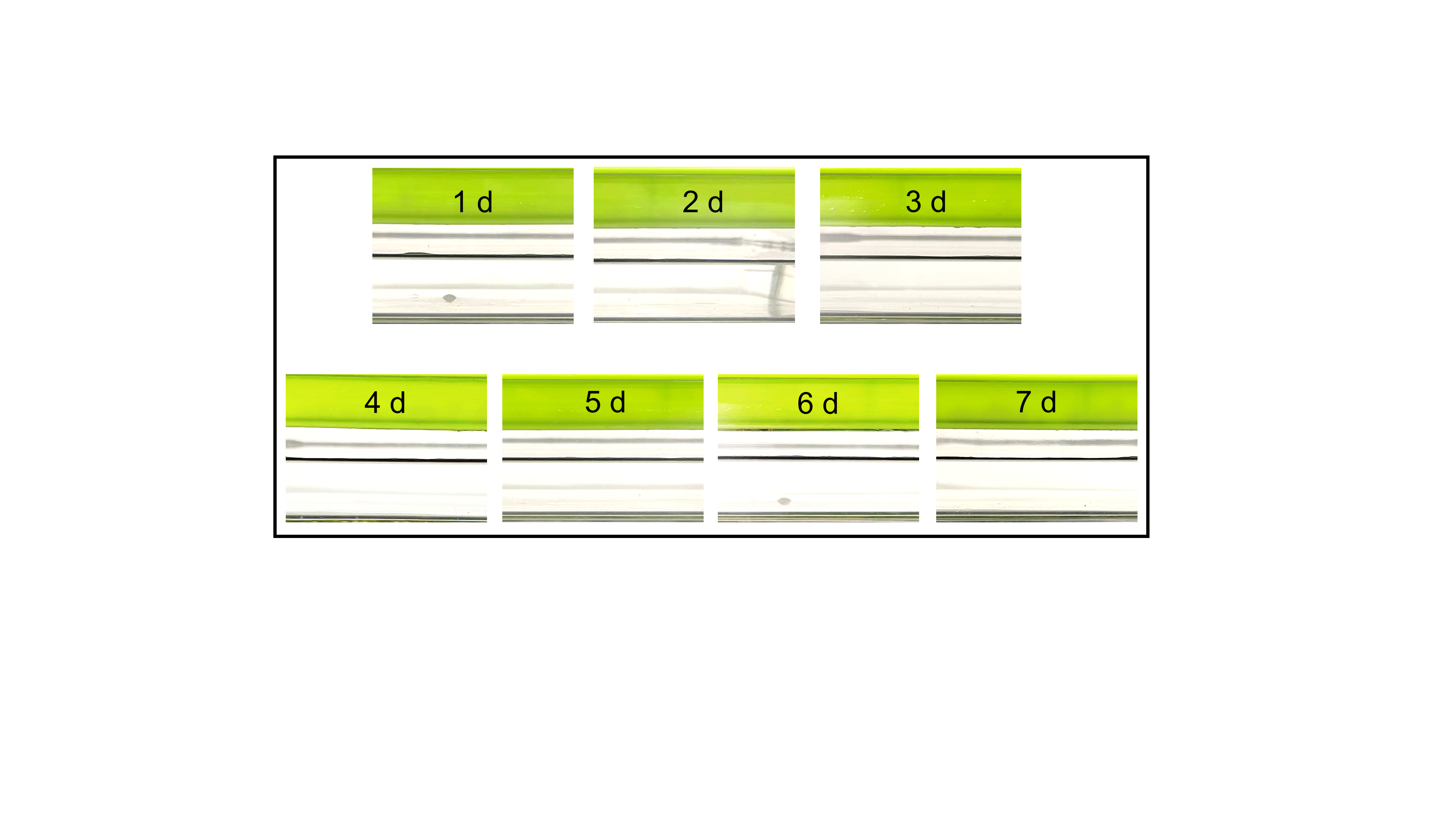


**Fig.** **S21** Optical photographs of the root system of wheat seeds irrigation with seawater for 7 d


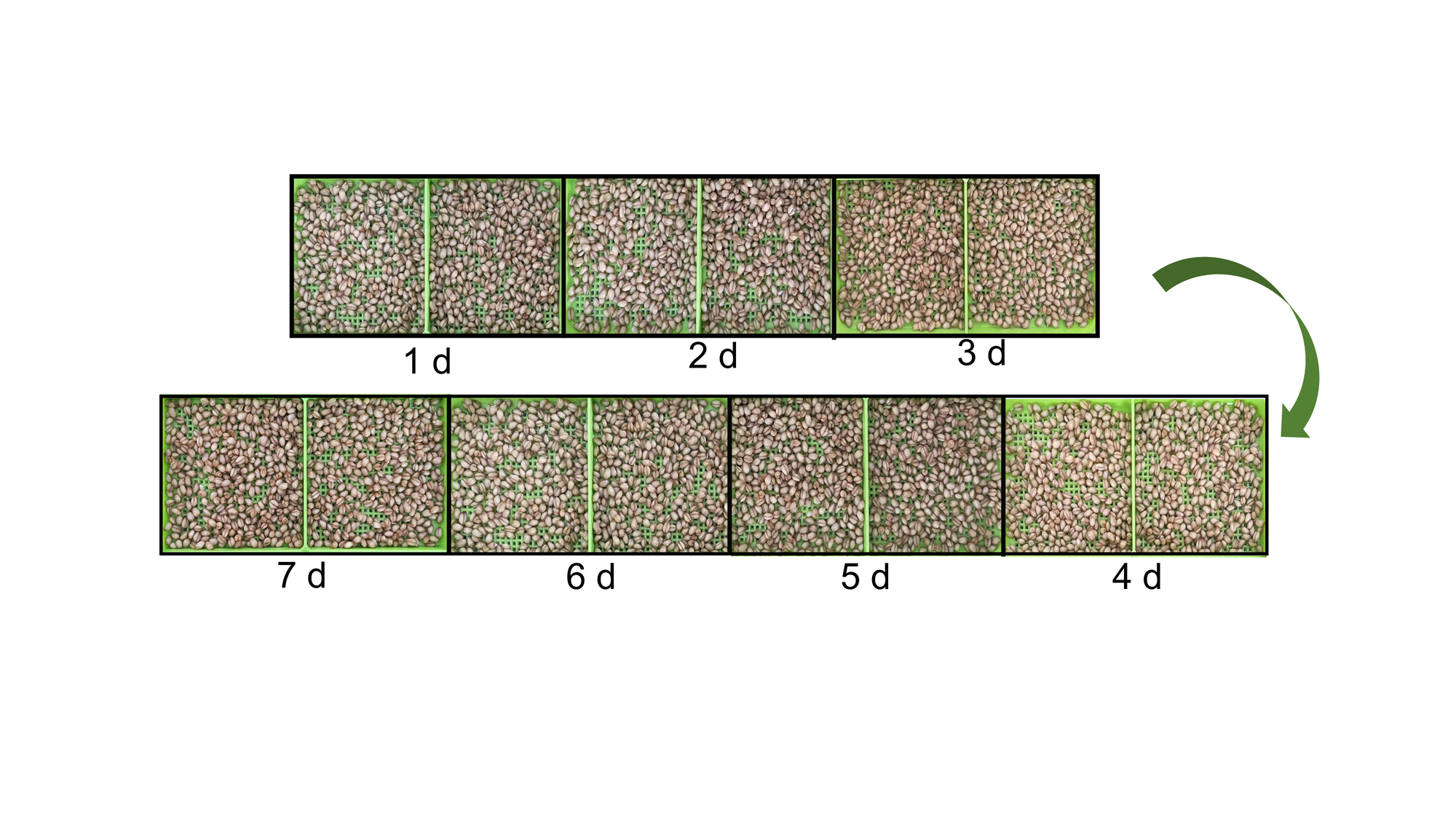


**Fig.** **S22** Optical images of wheat seeds irrigated with seawater for 7 d


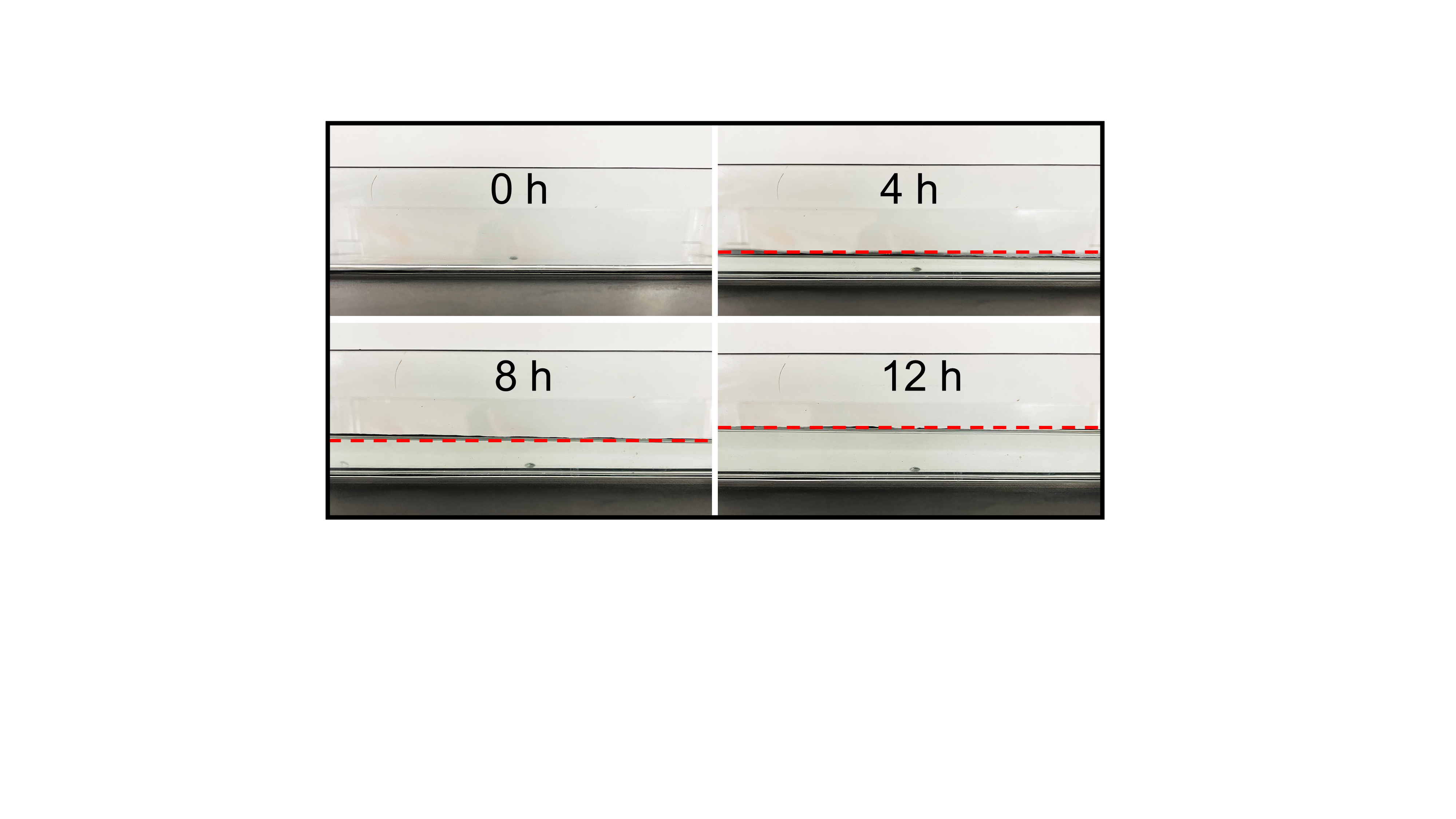


**Fig.** **S23** Optical images of the amount of drainage collected during 12 h of system operation, showing that the system drainage can meet the water demand for wheat cultivation


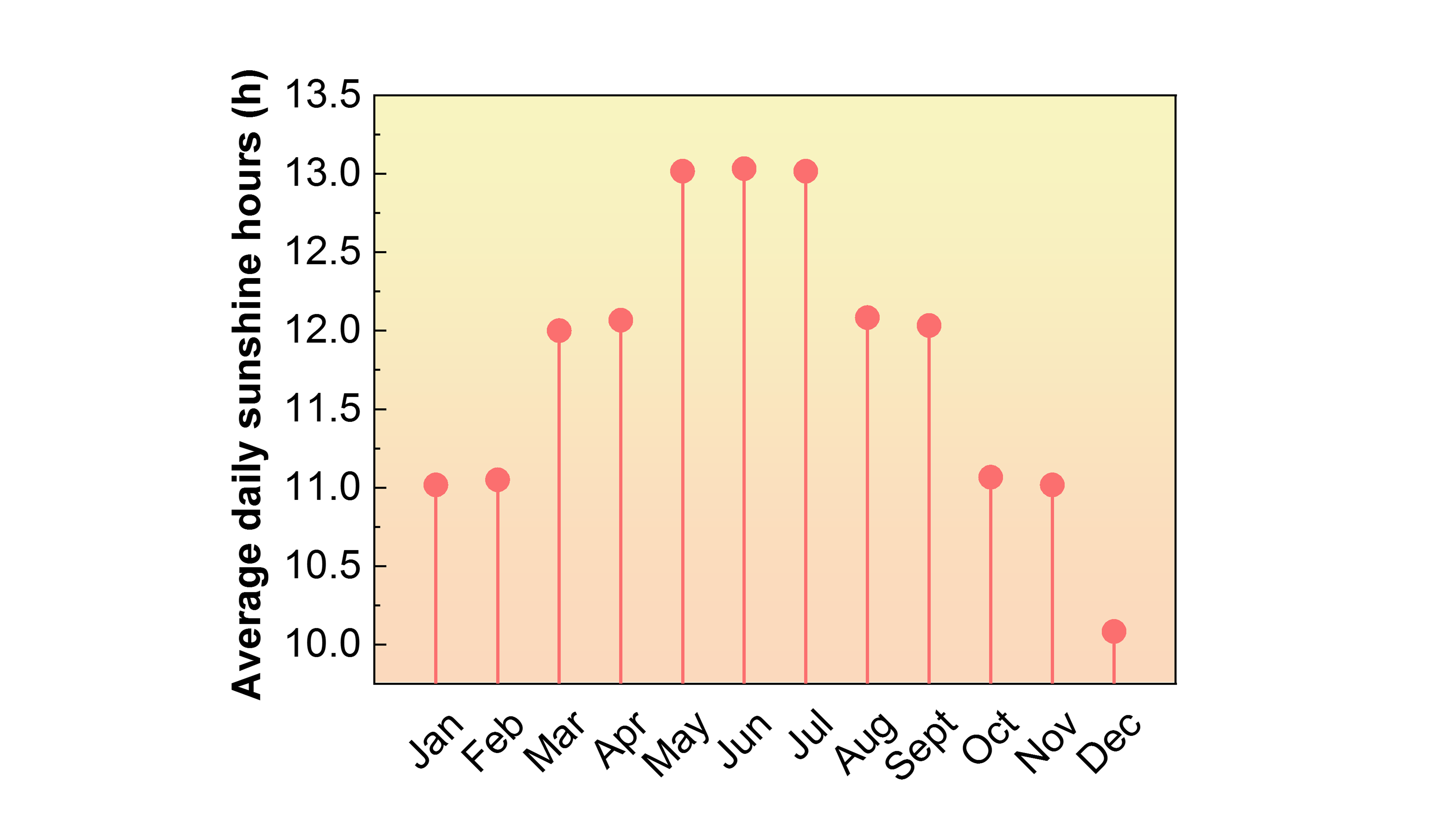


**Fig.** **S24** Monthly average daily sunshine duration over the selected region


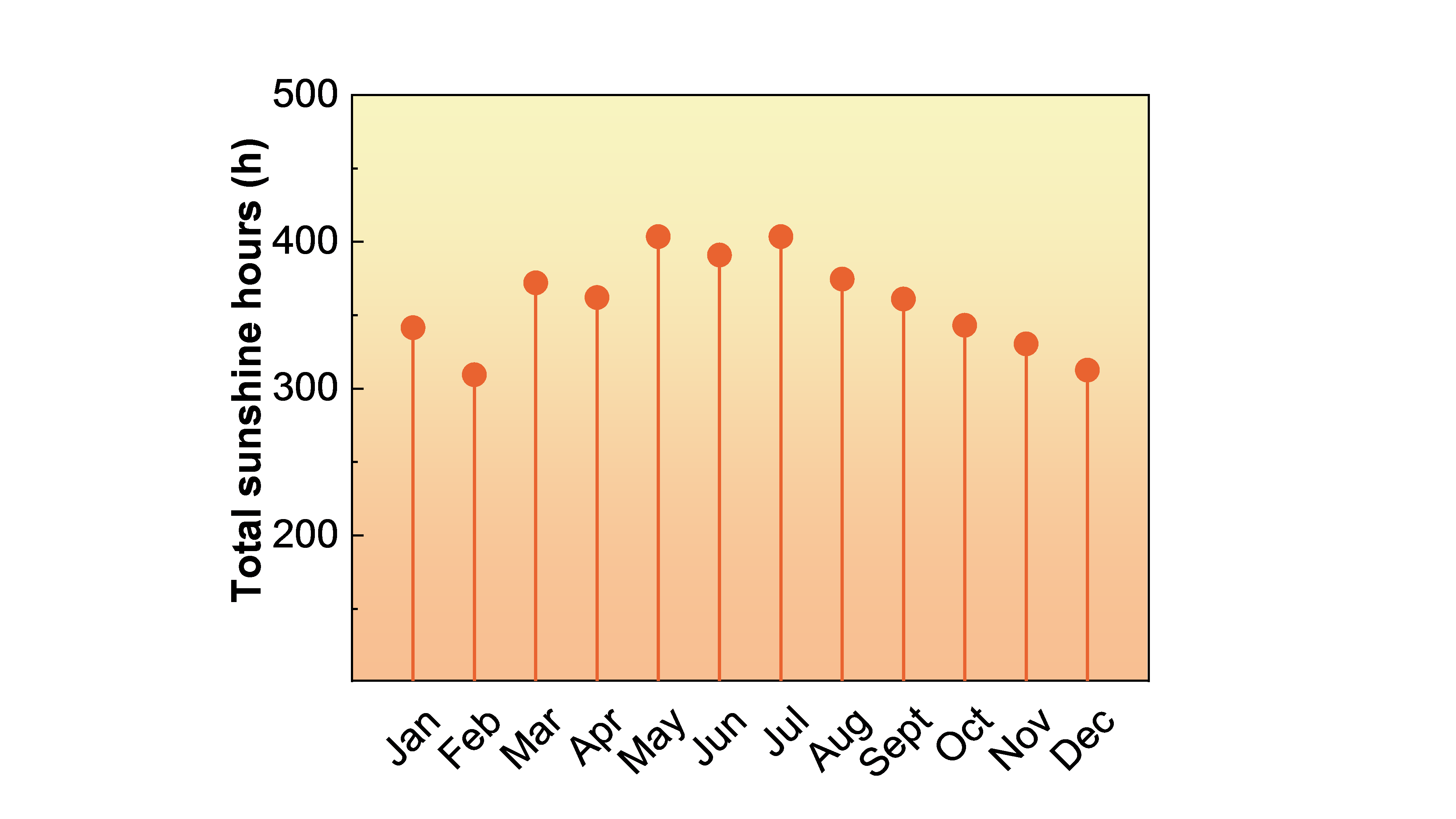


**Fig.** **S25** Monthly average total sunshine duration over the selected region


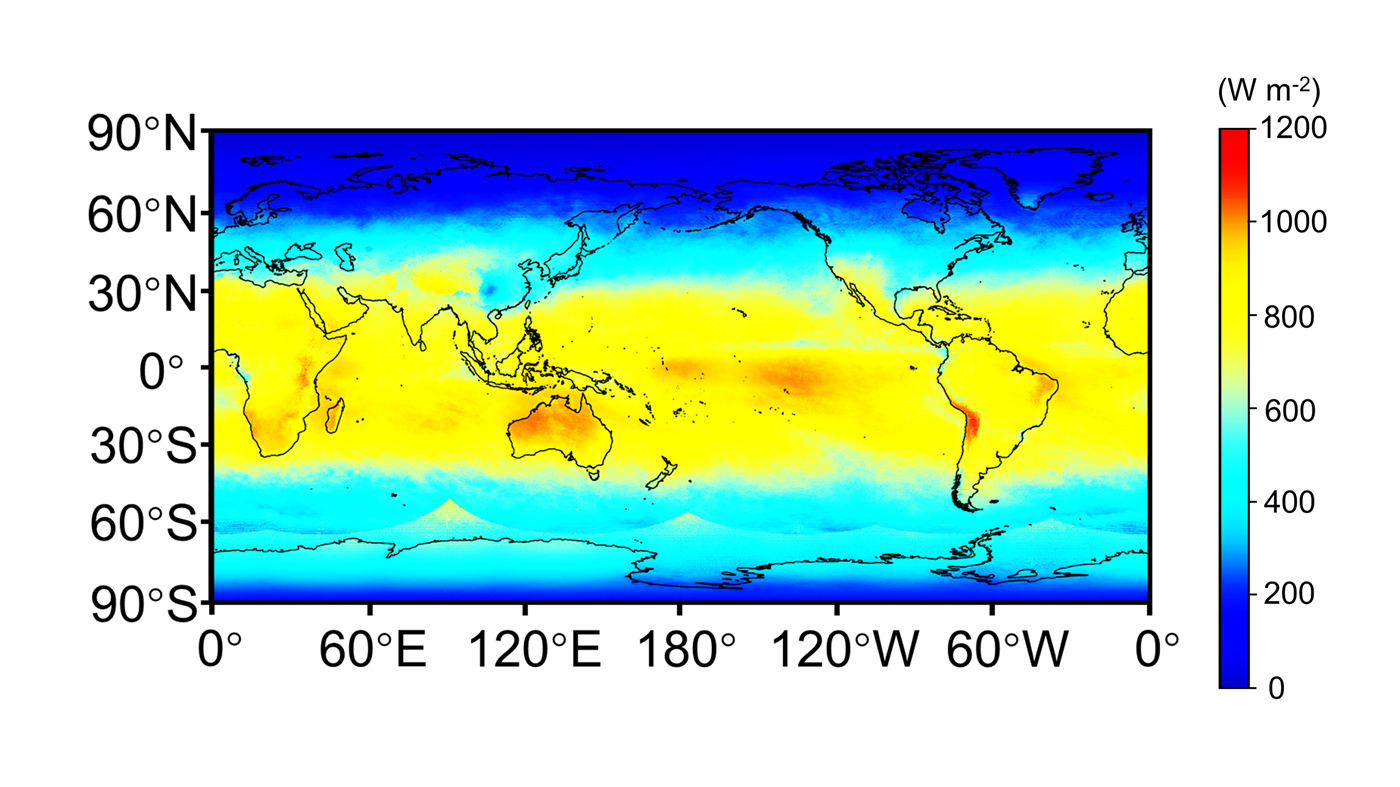


**Fig.** **S26** Global distribution of light intensity in spring


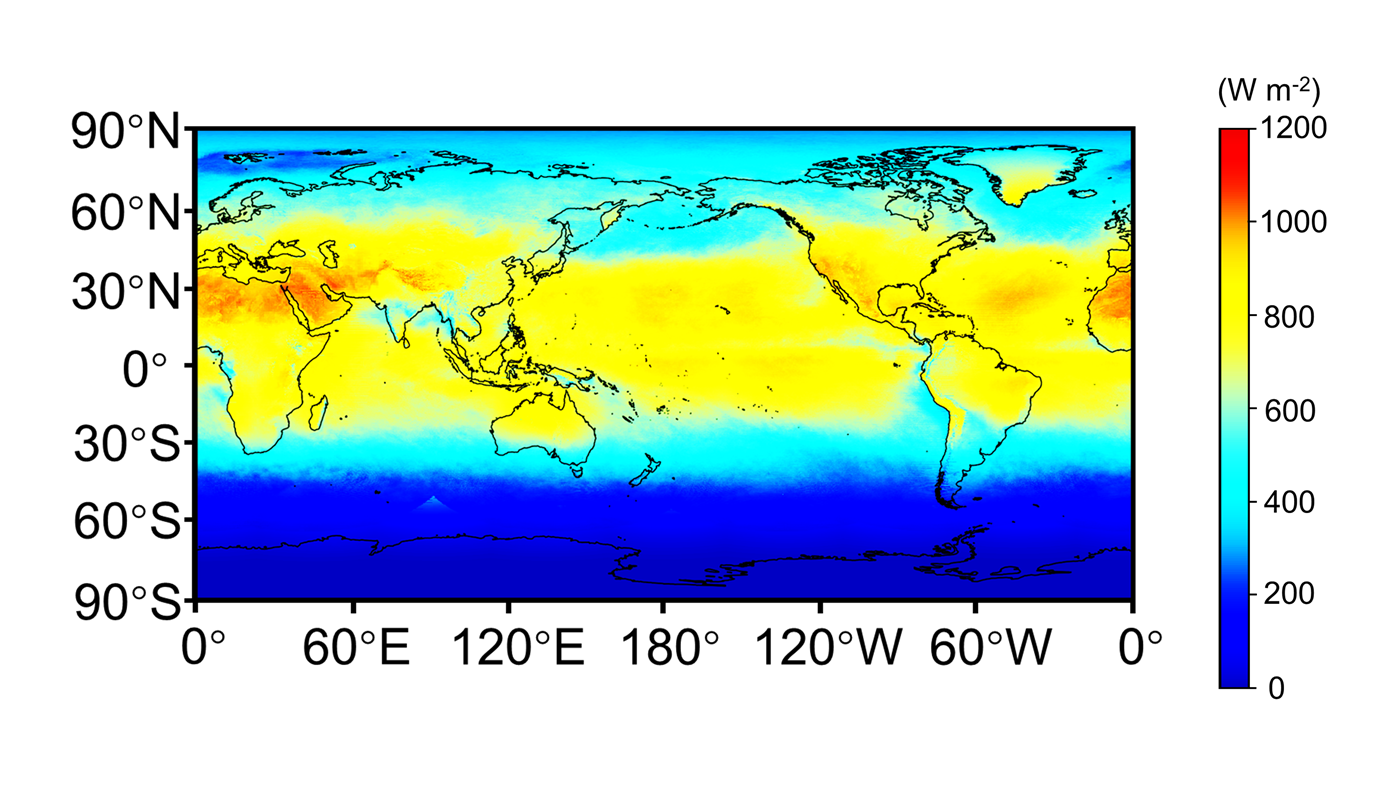


**Fig. S27** Global distribution of light intensity in Summer


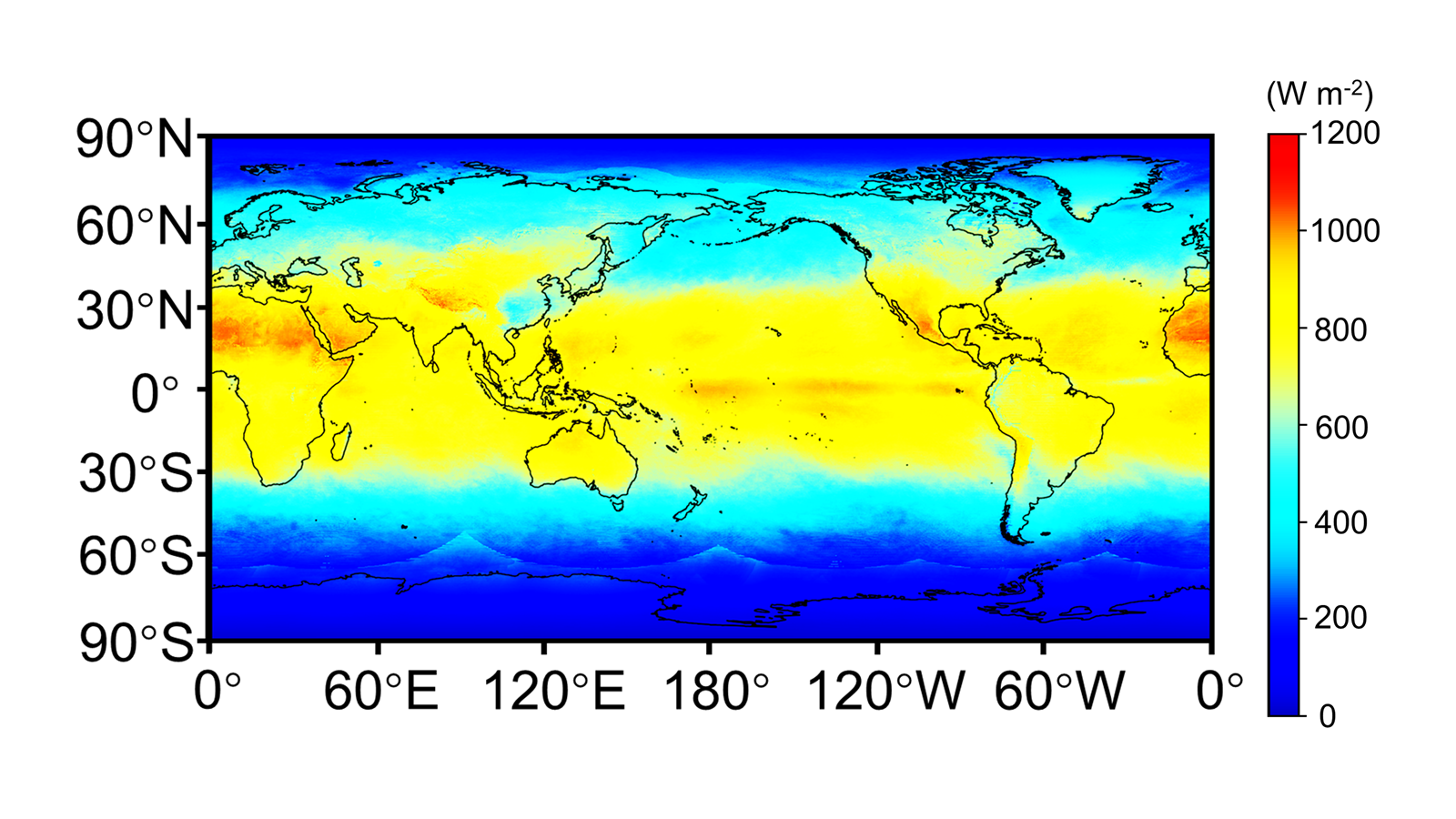


**Fig.** **S28** Global distribution of light intensity in Autumn


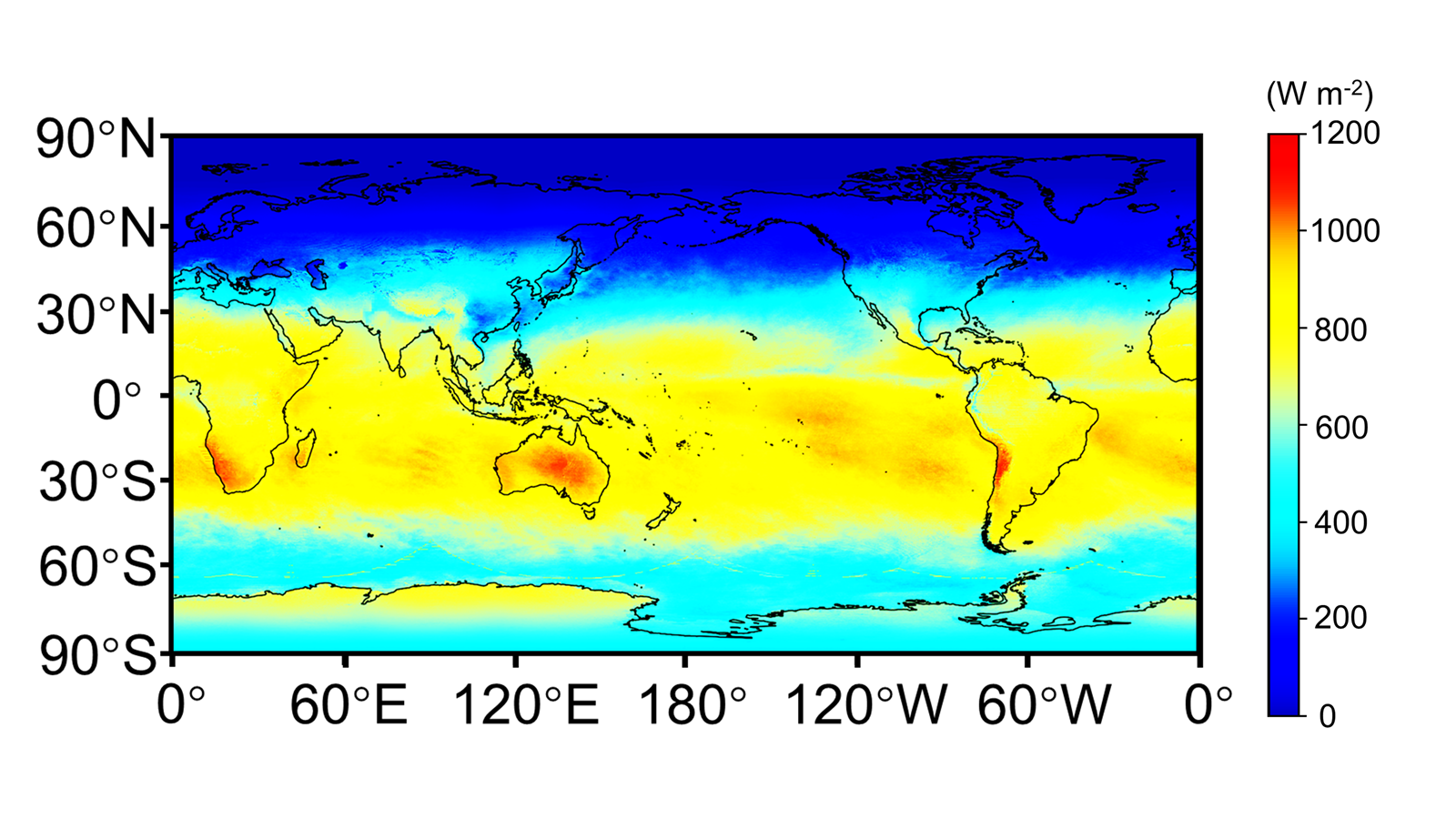


**Fig.** **S29** Global distribution of light intensity in Winter


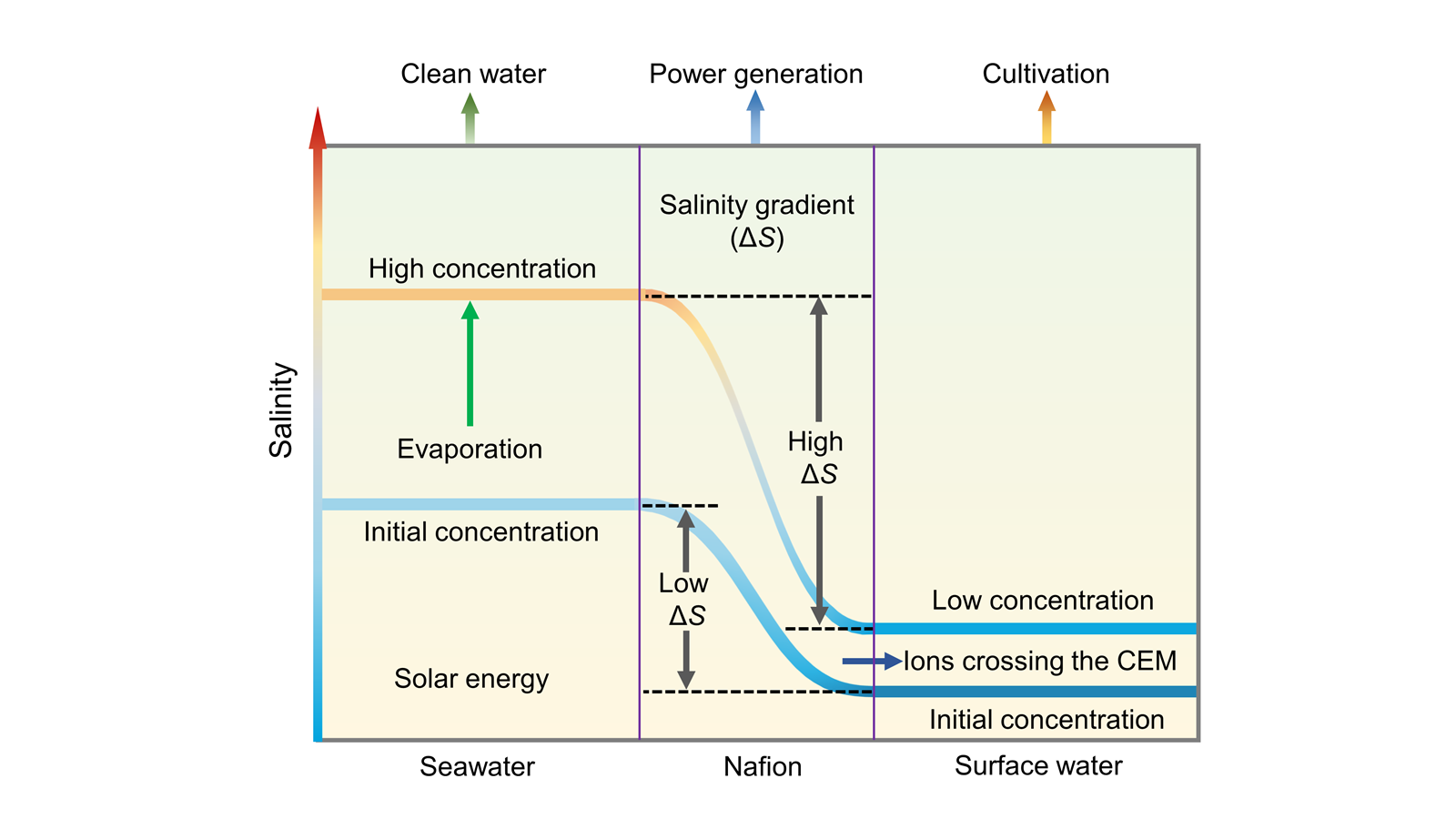


**Fig. S30** Energy flow diagram for desalination-generation-cultivation system


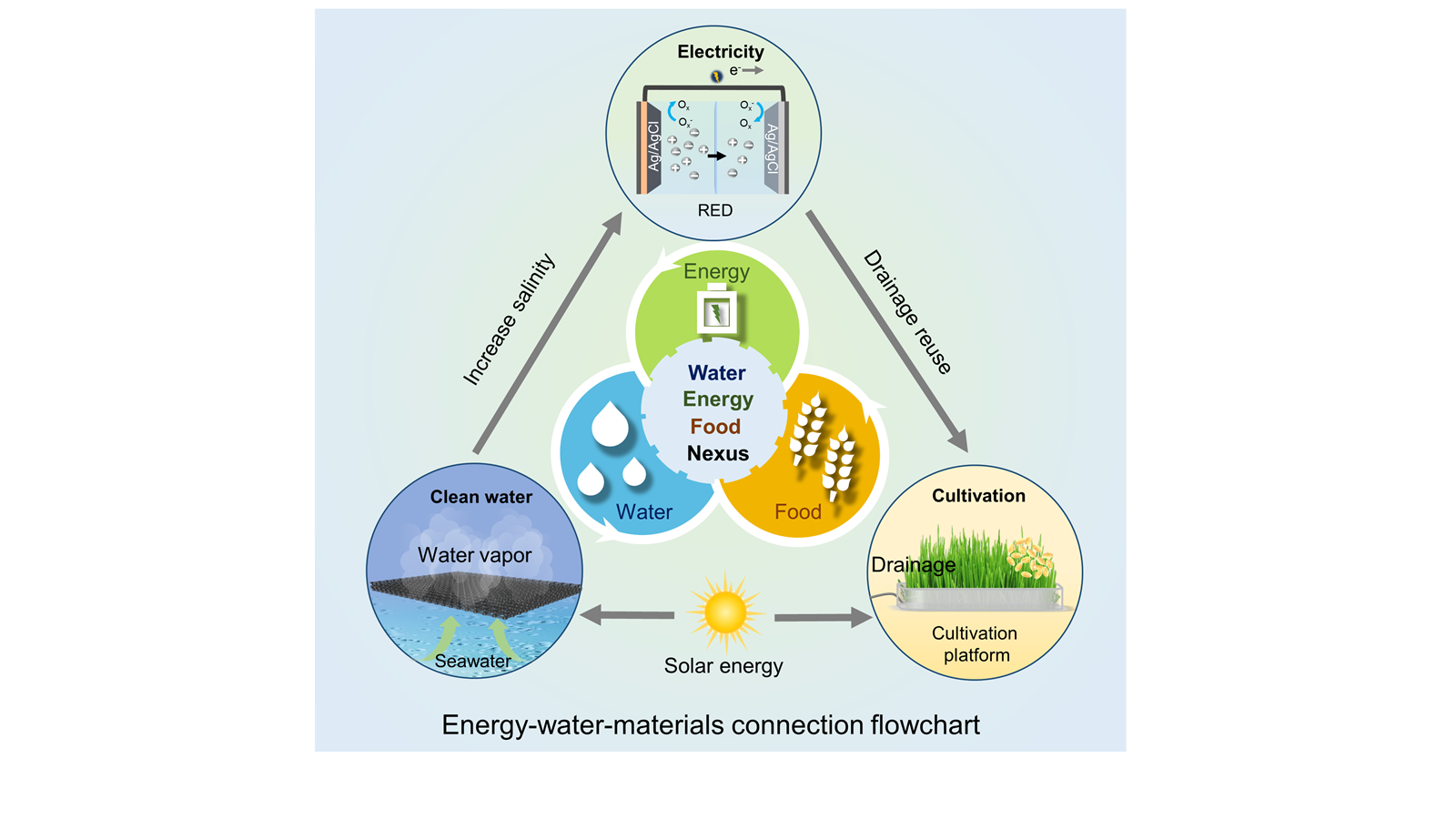


**Fig.** **S31** The energy-water-materials connection flowchart


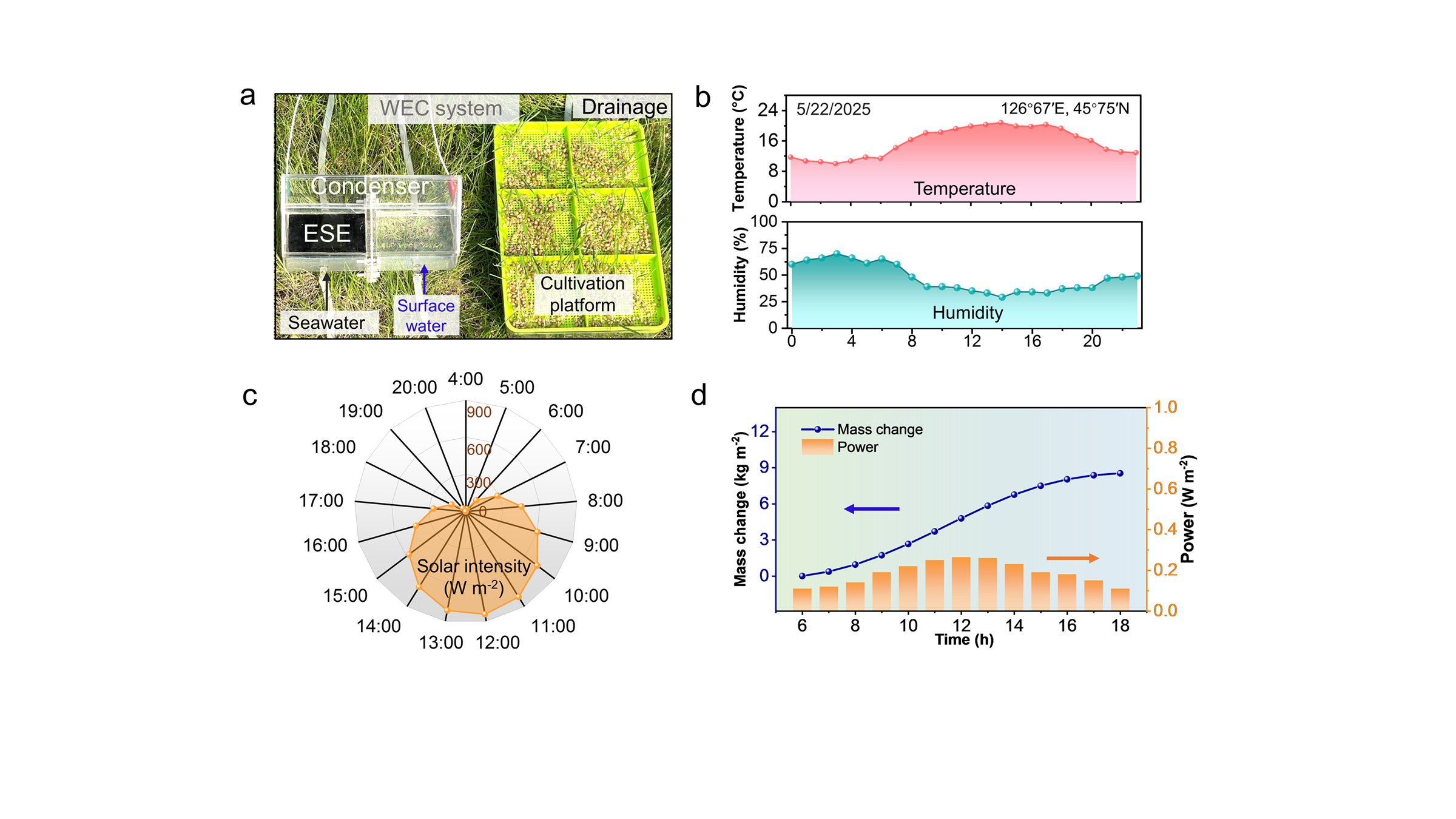


**Fig. S32** **a**) Practical outdoor operation of the WEC system. **b**) Trends in outdoor operational temperature and humidity variations. **c**) Solar intensity throughout the day during outdoor operation. **d**) Mass change and power output of the WEC during outdoor operation

**Supplementary References**

1. W. Tang, K. Yang, J. Qin, X. Li, X. Niu, A 16-year dataset (2000–2015) of high-resolution (3 h, 10 km) global surface solar radiation. Earth Syst. Sci. Data **11**(4), 1905–1915 (2019). <https://doi.org/10.5194/essd-11-1905-2019>
2. The People’s Government of Hainan Province. Notice of the Hainan Provincial People’s Government on the Issuance of the Hainan Carbon Peak Implementation Plan: Hainan Government [2022]. [https://doi.org/27. 00817365-1/2022-99466](https://doi.org/27.%2000817365-1/2022-99466)
3. Y. Tian, Y. Li, X. Zhang, J. Jia, X. Yang et al., Breath-Figure self-assembled low-cost Janus fabrics for highly efficient and stable solar desalination. Adv. Funct. Mater. **32**(33), 2113258 (2022). <https://doi.org/10.1002/adfm.202113258>
4. H. Liu, J. Gu, Y. Liu, L. Yang, L. Wang et al., Reconfiguration and self-healing integrated Janus electrospinning nanofiber membranes for durable seawater desalination. Nano Res. **16**(1), 489–495 (2023). <https://doi.org/10.1007/s12274-022-4733-4>
5. X. Dong, H. Li, L. Gao, C. Chen, X. Shi et al., Janus fibrous mats based suspended type evaporator for salt resistant solar desalination and salt recovery. Small **18**(13), 2107156 (2022). <https://doi.org/10.1002/smll.202107156>
6. J. Chen, J.L. Yin, B. Li, Z. Ye, D. Liu et al., Janus evaporators with self-recovering hydrophobicity for salt-rejecting interfacial solar desalination. ACS Nano **14**(12), 17419–17427 (2020). <https://doi.org/10.1021/acsnano.0c07677>
7. H.-H. Yu, L.-J. Yan, Y.-C. Shen, S.-Y. Chen, H.-N. Li et al., Janus poly(vinylidene fluoride) membranes with penetrative pores for photothermal desalination. Research **2020**, 3241758 (2020). <https://doi.org/10.34133/2020/3241758>
8. W. Xu, X. Hu, S. Zhuang, Y. Wang, X. Li et al., Flexible and salt resistant Janus absorbers by electrospinning for stable and efficient solar desalination. Adv. Energy Mater. **8**(14), 1702884 (2018). <https://doi.org/10.1002/aenm.201702884>
9. X. Wang, Z. Li, S. Xing, W. Kuang, C. Dong et al., Toward multitasking solar desalination: a Janus and scalable paper evaporator with light trapping, heat confinement, salt resistance, and pollutant degradation. J. Mater. Chem. A **11**(19), 10287–10296 (2023). <https://doi.org/10.1039/D2TA09653F>
10. M. Xie, P. Zhang, Y. Cao, Y. Yan, Z. Wang et al., A three-dimensional antifungal wooden cone evaporator for highly efficient solar steam generation. NPJ Clean Water **6**, 12 (2023). <https://doi.org/10.1038/s41545-023-00231-3>
11. L. Hou, N. Wang, L.-J. Yu, J. Liu, S. Zhang et al., High-performance Janus solar evaporator for water purification with broad spectrum absorption and ultralow heat loss. ACS Energy Lett. **8**(1), 553–564 (2023). <https://doi.org/10.1021/acsenergylett.2c02567>
12. Y. Kuang, C. Chen, S. He, E.M. Hitz, Y. Wang et al., A high-performance self-regenerating solar evaporator for continuous water desalination. Adv. Mater. **31**(23), 1900498 (2019). <https://doi.org/10.1002/adma.201900498>
13. X. Chen, S. He, M.M. Falinski, Y. Wang, T. Li et al., Sustainable off-grid desalination of hypersaline waters using Janus wood evaporators. Energy Environ. Sci. **14**(10), 5347–5357 (2021). <https://doi.org/10.1039/D1EE01505B>
14. M. Molinos-Senante, R. Sala-Garrido, Evaluation of energy performance of drinking water treatment plants: Use of energy intensity and energy efficiency metrics. Appl. Energy **229**, 1095–1102 (2018). <https://doi.org/10.1016/j.apenergy.2018.08.102>
15. M. Molinos-Senante, A. Maziotis, R. Sala-Garrido, M. Mocholí-Arce, Estimating the cost efficiency and marginal cost of carbon reductions in the production of drinking water. Sustain. Cities Soc. **85**, 104091 (2022). <https://doi.org/10.1016/j.scs.2022.104091>
16. M. Molinos-Senante, C. Guzmán, Reducing CO_2_ emissions from drinking water treatment plants: a shadow price approach. Appl. Energy **210**, 623–631 (2018). <https://doi.org/10.1016/j.apenergy.2016.09.065>
17. L. Zib, D.M. Byrne, L.T. Marston, C.M. Chini, Operational carbon footprint of the U.S. water and wastewater sector’s energy consumption. J. Clean. Prod. **321**, 128815 (2021). <https://doi.org/10.1016/j.jclepro.2021.128815>
